# *Anopheles* metabolic proteins in malaria transmission, prevention and control: a review

**DOI:** 10.1186/s13071-020-04342-5

**Published:** 2020-09-10

**Authors:** Eunice Oluwatobiloba Adedeji, Olubanke Olujoke Ogunlana, Segun Fatumo, Thomas Beder, Yvonne Ajamma, Rainer Koenig, Ezekiel Adebiyi

**Affiliations:** 1 Covenant University Bioinformatics Research (CUBRe), Covenant University, Ota, Ogun State Nigeria; 2 Department of Biochemistry, Covenant University, Ota, Ogun State Nigeria; 3 Department of Non-Communicable Disease Epidemiology, London School of Hygiene & Tropical Medicine, Keppel St, Bloomsbury, London, UK; 4 Integrated Research and Treatment Center, Center for Sepsis Control and Care (CSCC), Jena University Hospital, Am Klinikum 1, 07747 Jena, Germany; 5 Computer and Information Sciences, Covenant University, Ota, Ogun State Nigeria; 6 Division of Applied Bioinformatics, German Cancer Research Center (DKFZ), G200, Im Neuenheimer Feld 280, 69120 Heidelberg, Germany

**Keywords:** Immune response, Insecticide, Insecticide resistance, *Plasmodium*, Vector control, Acetylcholinesterase

## Abstract

The increasing resistance to currently available insecticides in the malaria vector, *Anopheles* mosquitoes, hampers their use as an effective vector control strategy for the prevention of malaria transmission. Therefore, there is need for new insecticides and/or alternative vector control strategies, the development of which relies on the identification of possible targets in *Anopheles*. Some known and promising targets for the prevention or control of malaria transmission exist among *Anopheles* metabolic proteins. This review aims to elucidate the current and potential contribution of *Anopheles* metabolic proteins to malaria transmission and control. Highlighted are the roles of metabolic proteins as insecticide targets, in blood digestion and immune response as well as their contribution to insecticide resistance and *Plasmodium* parasite development. Furthermore, strategies by which these metabolic proteins can be utilized for vector control are described. Inhibitors of *Anopheles* metabolic proteins that are designed based on target specificity can yield insecticides with no significant toxicity to non-target species. These metabolic modulators combined with each other or with synergists, sterilants, and transmission-blocking agents in a single product, can yield potent malaria intervention strategies. These combinations can provide multiple means of controlling the vector. Also, they can help to slow down the development of insecticide resistance. Moreover, some metabolic proteins can be modulated for mosquito population replacement or suppression strategies, which will significantly help to curb malaria transmission.
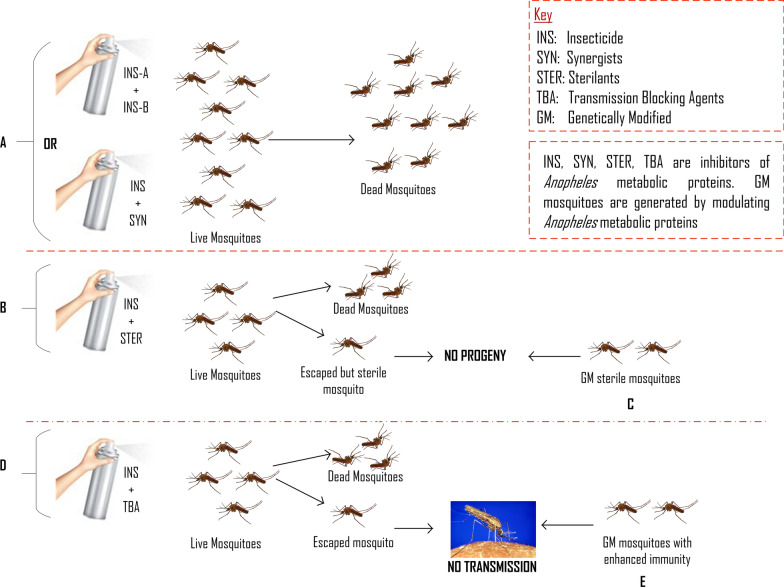

## Background

Malaria remains a universal health challenge affecting over 200 million of the world’s population annually. Although malaria burden is highest in Africa (93% of malaria cases), a global incidence rate of 57 cases per 1000 population has been reported annually between 2014–2018 [1]. Malaria is an infectious disease caused by the parasite *Plasmodium* and transmitted by female *Anopheles* mosquitoes, which vary from one region to another [2, 3]. The major *Anopheles* species include *An. gambiae*, *An. stephensi*, *An. dirus*, *An. coluzzii*, *An. albimanus*, *An. funestus* and *An. arabiensis* amongst others. Transmission of *Plasmodium* depends on the completion of its developmental cycle in the mosquito, a process that occurs alongside the digestion of the blood meal and egg development in the mosquito [4]. This blood meal is crucial for oogenesis [5]. Hence, *Anopheles* mosquito’s ability to transmit malaria is directly linked to its ability to feed on and digest a blood meal from a malaria-infected person [6]. These processes i.e. blood digestion, egg development and parasite development in the mosquito occur simultaneously and are tightly linked to metabolism. Metabolism refers to all the enzyme-catalyzed chemical transformations that occur in the cell of an organism [7] and metabolic proteins consist of enzymes as well as transporters. Since metabolism is substantial for the survival and proper functioning of an organism, metabolic proteins provide a good biological space to serve as vector control targets.

**Fig. 1:**
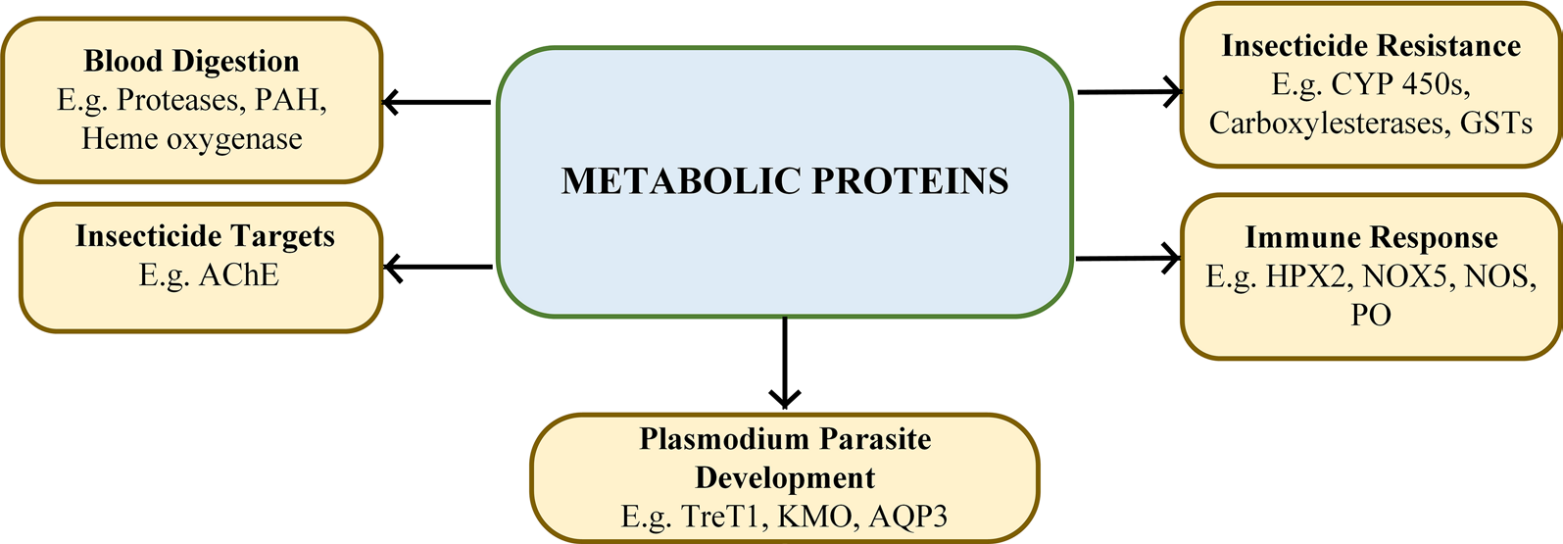
Role of *Anopheles* metabolic proteins in malaria transmission and control. *Abbreviations*: PAH, phenylalanine-4-hydroxylase; AChE, acetylcholinesterase; CYP 450s, cytochrome P450s; AQP3, aquaporin 3; GSTs, glutathione S-transferases; HPX2, heme peroxidase 2; NOX5, NADPH oxidase 5; NOS, nitric oxide synthase; PO, phenoloxidase; TreT1, trehalose transporter; KMO, kynurenine 3-monooxygenase; CEs, carboxylesterases

Interestingly, some metabolic proteins involved in digesting ingested blood, absorbing nutrients and oogenesis, also play a role in the development of *Plasmodium* in the mosquito [8]. For example, trypsin produced in *Anopheles* midgut might activate *Plasmodium* chitinase that allows the parasite to evade physical barriers in the mosquito [9]. In addition, ingestion of the parasite by *Anopheles* triggers an innate immune response in the mosquito to circumvent parasite development [10]. This immune response is a cascade of reactions involving some metabolic proteins of the mosquito. This interplay suggests the importance of metabolic proteins in *Plasmodium* development in the mosquito and consequently malaria transmission. Aside from being involved in blood digestion and parasite development, *Anopheles* metabolic proteins such as the acetylcholinesterase (AChE) are, also important targets for vector control strategies [11]. Figure 1 gives a schematic overview of the contributions of the metabolic proteins of *Anopheles* mosquitoes in malaria transmission and control.

In the past decades, vector control greatly depended on the use of insecticides for indoor residual spraying (IRS) and insecticide-treated nets (ITNs) [12]. These strategies greatly reduced malaria deaths between 2010 and 2015, as 50% reduction in malaria deaths was reported and 79% of this reduction was attributed to insecticide use [13]. Some insecticides target metabolic proteins. For instance, AChE has been the only target of organophosphate and carbamate insecticides for many years [14]. In addition, AChE is the target for temephos and fenthion that are organophosphate insecticides and target the larval stage of mosquitoes, and thus, are used in larviciding strategies [15]. These larvicides, upon application to breeding sites of mosquitoes, prevent their further development into adult forms, consequently, reducing adult mosquito population density and ultimately decreasing malaria transmission rates [16]. Meanwhile, the toxicity of many of the currently available insecticides to non-target species and the ever increasing resistance of *Anopheles* to commonly used classes of insecticides, necessitate the identification of novel targets for vector control [17–21]. Also, metabolic resistance to insecticides is mediated by the activities of detoxifying enzymes [22] and there is evidence that combining insecticides with the inhibitors of these enzymes can considerably reduce insecticide resistance [23]. Therefore, the modulation of metabolic proteins provides a plethora of potential intervention strategies.

Since *Anopheles* metabolic proteins perform many crucial functions that contribute to malaria transmission and control, a critical review of their roles can provide insights into the possibilities of utilizing *Anopheles* metabolic proteins for more targeted vector control strategies. Therefore, this review summarizes the information on the role of *Anopheles* metabolic proteins in the transmission and control of malaria as well as gives insights into future targeted vector control strategies. The role of metabolic proteins is discussed under the following headings: insecticide target; resistance to insecticides; blood digestion; immune response; and *Plasmodium* development in the mosquito, and their manipulation for vector control strategies.

## Metabolic proteins as insecticide targets

Insecticides are crucial for controlling the malaria vector and consequently, preventing malaria transmission. The four main classes of insecticides used for both indoor and outdoor spraying are organophosphates, organochlorides, carbamates and pyrethroids. Pyrethroids are used in ITNs because of their insecticidal potency and relative safety for domestic use [24]. Many of the known insecticides act on proteins that mediate neuronal processes. Examples of these insecticide targets are AChE, gamma-aminobutyric acid (GABA)-chloride ionophore complexes, sodium ion channels [25–27]. However, out of the four classes of insecticides employed for malaria control program, only carbamates and organophosphates target a metabolic protein.

Organophosphate and carbamate insecticides competitively inhibit AChE (EC 3.1.1.7), an enzyme that hydrolyzes acetylcholine into acetate and choline [25, 28]. This hydrolysis reaction terminates the transmission of the cholinergic neuronal signal after an excitation signal [29]. Inhibition of this reaction results in continuous stimulation of the nervous system and consequently leads to the death of the mosquito [30]. These insecticides elicit their inhibitory effects by forming a covalent bond with the catalytic serine residue of AChE [25]. Most insects, including *Anopheles*, have two AChE genes, *ace1* (AChE1) and *ace2* (AChE2) [31, 32]. However, AChE1 is the major nervous system cholinesterase in many of these insects and experimental evidence exists showing that AChE1 hydrolyzes most acetylcholine in *An. gambiae* [33]. Thus, AChE1 is the target for carbamates and organophosphates in *Anopheles* species.

A study comparing the effect of inhibiting the two AChE genes in *Tribolium castaneum* (TcAChE1 and TcAChE2) revealed that while the inhibition of TcAChE1 resulted in mortality, inhibition of TcAChE2 by RNAi led to a reduction in egg-laying and hatching, and retarded insect development [34]. Similarly in *An. gambiae*, AChE1 is the major AChE insecticide target while AChE2 was suggested to perform some other biological roles other than cholinergic functions [33]. Therefore, *An. gambiae* AChE2 may play a role in reproduction and development of the mosquito. Further research is necessary to confirm these as AChE2 may be a potential target for manipulation or inhibition to achieve population suppression of mosquitoes.

In general, carbamate and organophosphate insecticides are very important classes of insecticides as they have been considered as alternatives for use in ITNs [11]. However, like most insecticide classes, resistance to carbamates are increasingly reported in *Anopheles*. Hence, organophosphates remain the main class of insecticide used for IRS or resistance management by the National Malaria Control Programmes in most African countries [35, 36]. In some recent studies, 100% susceptibility to organophosphate insecticides in *An. gambiae* and *An. funestus* was observed [37, 38], emphasizing the importance of AChE as a crucial target for malaria vector control strategies.

**Fig. 2:**
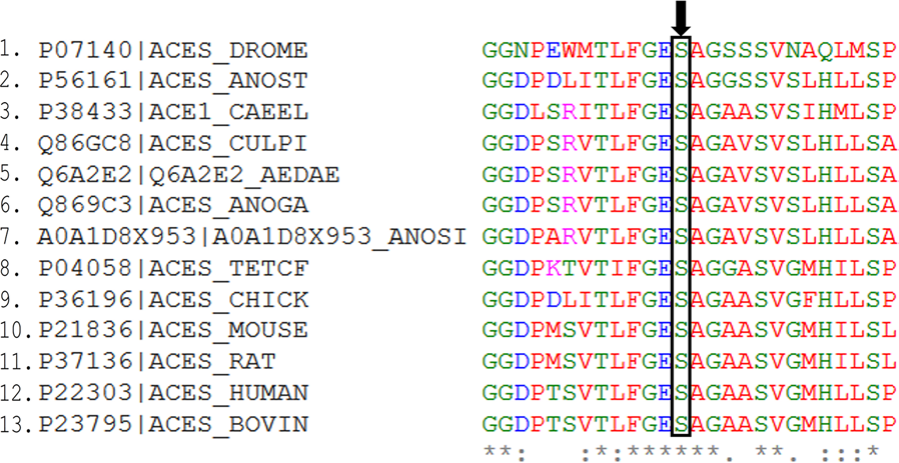
Conserved catalytic serine residue in acetylcholinesterase (AChE) targeted by insecticides in diverse organisms. The sequences shown are from *Drosophila melanogaster* (DROME), *Tetronarce californica* (TETCF), *Mus musculus* (MOUSE), *Homo sapiens* (HUMAN), *Bos taurus* (BOVIN), *Rattus norvegicus* (RAT), *Caenorhabditis elegans* (CAEEL), *An. stephensi* (ANOST), *An. gambiae* (ANOGA), *Culex pipiens* (CULPI), *An. sinensis* (ANOSI), *Aedes aegypti* (AEDAE). The name of each organism starts with its UniProt accession number. Conserved catalytic serine is shown by a black arrow. The catalytic serine residue is conserved across insects (1–2 and 4–7), mammals (10–13), birds (9), nematode (3) and fish (8). * indicates positions that have single and conserved amino acid residues; : indicates conservation between amino acid residues of strongly similar properties; . indicates conservation between amino acid residues of weakly similar properties

**Fig. 3:**
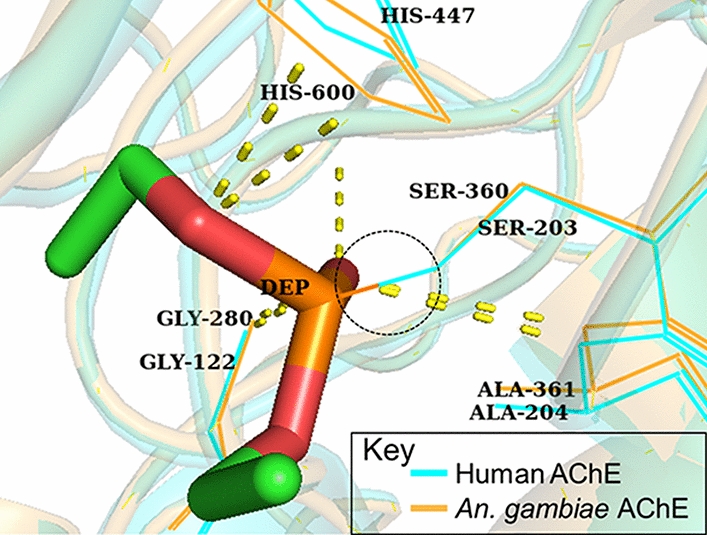
Paraoxon binds conserved residues in both humans’ and mosquitoes’ acetylcholinesterase (AChE), hence toxic to humans. Diethyl phosphonate (DEP) from paraoxon binds covalently to Ser203 in humans AChE (hAChE; PDB ID: 5hf5) and Ser360 in *An. gambiae* AChE (AgAChE; PDB ID: 5x61), thus inhibiting them. Covalent binding is highlighted in black dotted circle. Also, DEP interacts with Gly122, His447 and Ala204 in hAChE, Gly280, His600 and Ala361 in AgAChE through hydrogen bonds

Aside from insecticide resistance evolving in *Anopheles*, another major concern with currently available insecticides is their toxicity to non-target species [39]. This is because most insecticides inhibit proteins that are generally conserved across species in a non-specific manner. For example, organophosphates such as paraoxon are irreversible inhibitors of AChE, mediating their action by phosphorylating the highly conserved catalytic serine residue in AChE [40]. The conservation of AChE catalytic serine residue was examined in 13 animal species using Clustal Omega version 1.2.4 [41] on the European Molecular Biology Laboratory-European Bioinformatics Institute’s (EMBL-EBI) platform for multiple sequence alignment (MSA) [42] (Additional file 1: Figure S1). It was observed that this catalytic serine residue is conserved across all the species of animals that included insects, mammals, birds, nematode and fish (Fig. 2). In particular, the inhibition of AChE by paraoxon, an organophosphate insecticide, results in covalent attachment of a diethyl phosphonate (DEP) to the side-chain of the catalytic serine. To compare the interaction of DEP with human AChE (hAChE) and *An. gambiae* AChE (AgAChE), DEP-bound hAChE (PDB ID: 5hf5) [43] was aligned to AgAChE (PDB ID: 5x61) [44] using PyMOL [45] (Fig. 3). Both hAChE and AgAChE interacted with DEP by binding conserved residues; DEP binds catalytic serine 203 in hAChE and catalytic serine 360 in AgAChE through covalent interaction. Additionally, DEP interacts with Gly122, His447 and Ala204 in hAChE as well as their conserved counterparts, i.e. Gly280, His600 and Ala361 in AgAChE through hydrogen bonds. Although organophosphate insecticides are irreversible inhibitors of AChE, carbamates are its reversible inhibitors that carbamylate its catalytic serine residue [14]. Therefore, these insecticides inhibit AChEs in non-target species and cause severe toxicity problems.

Since most of these insecticides target the nervous system, and children are more susceptible to insecticide toxicity, neurotoxicity in children due to insecticides exposure is of increasing concern [18, 46]. To address this problem of toxicity, more specific insecticides have to be developed for known insecticide targets or newly identified ones. Some studies have identified inhibitors that have greater selectivity for AgAChE than hAChE. An example of this was reported in a study in which differential high throughput screening (HTS) of several compounds for selective inhibition of AgAChE was carried out [47]. One of the selective inhibitors identified in their study was a phenoxyacetamide-based inhibitor that was 100-times more selective for AgAChE than for hAChE. In another study, Carlier et al. [48] screened some alkyl chemically substituted 1-alkylpyrazol-4-yl methylcarbamate compounds for their selective inhibition of AChE. They identified three compounds, namely, cyclopentylmethyl pyrazol-4-yl methyl carbamate, cyclobutylmethyl pyrazol-4-yl methyl carbamate and 3-methylbutyl- pyrazol-4-yl methyl carbamate that were 250, 120 and 96 times, respectively, more selective for AgAChE than for hAChE [48]. These two studies suggest that *Anopheles* AChE can be selectively inhibited by more specific insecticides. Further studies towards identifying these selective inhibitors that could serve as novel insecticides are possible since the crystal structure of the catalytic domain of AgAChE is available [44]. Still, a new insecticide target should ideally be a protein that is important for the survival of target species and unique to them (i.e. absent in non-target species). Different studies have suggested some metabolic proteins that can serve as possible insecticide targets, namely, carbonic anhydrases, arylalkylamine N-acetyltransferases, V-ATPase and phosphofructokinase (PFK) [49–52]. These potential insecticide targets are further discussed in the subsection “Identifying novel insecticide targets”.

In addition to being targets for adulticides, metabolic proteins are targets for larvicides. For example, temephos is an organophosphate larvicide targeting AChE. Another group of larvicides, benzoylurea (BFU), inhibits chitin biosynthesis by targeting chitin synthase 1 (CHS1: EC 2.4.1.16) [53]. Chitin biosynthesis is essential for insect growth; therefore, its inhibitors are insect growth regulators, affecting the growth and survival of insects. One of the most effective larvicides currently available is diflubenzuron (DFB), a BFU, which is used in the control of *Culex pipiens* [54]. Novaluron, another CHS1 inhibitor is effective against *Aedes aegypti*, inhibiting adult emergence by at least 70% [55]. In a study by Zhang et al. [56], exposure of third-instar larvae of *An. gambiae* to 50 μg/l DFB resulted in about 60% mortality in 48 h. Although in their study, DFB had minimal *in vitro* inhibition on *An. gambiae* CHS1 and no *in vivo* inhibition on *An. gambiae* pupae, a different study showed that knockdown of CHS1 by RNAi in *An. gambiae* increased toxicity to DFB in the mosquito [57]. These studies suggest that CHS1 can be targeted for vector control strategies. Further studies investigating the inhibitory effect of BFUs on CHS1 in *Anopheles* and the exposure effect of other larval stages to BFUs can guide the use of the larvicides in malaria control.

## Metabolic proteins and insecticide resistance

The intense use of the few available insecticide classes for IRS and ITNs has resulted in increased resistance in mosquitoes [21]. Insecticide resistance in *Anopheles* has been reported for all the four main classes of insecticides being used in malaria control [1]. In 2017, Riveron et al. [58] observed high resistance to permethrin (a pyrethroid insecticide) and dichlorodiphenyltrichloroethane (DDT) in *An. gambiae* with no significant mortalities after exposing the mosquitoes to these insecticides for 6 h. Their study corroborated earlier reports on the development of DDT and pyrethroid resistance in *Anopheles* [59–62]. The two most characterized insecticide resistance mechanisms are metabolic resistance and target-site resistance [63]. In addition to these, there are three other insecticide resistance mechanisms, namely, behavioral resistance, cuticular resistance, and sequestration by the chemosensory proteins sensory appendage protein 2 (SAP2) [64, 65]. Understanding these mechanisms is important in guiding decisions on resistance management strategies [66].

### Metabolic resistance

Metabolic resistance to insecticides is caused by changes in the sequestration, transport and detoxification of insecticides and their metabolites [66]. Insecticides are xenobiotics (i.e. foreign to the body); thus, *Anopheles* xenobiotics detoxifying enzymes break down insecticides into less harmful substances, hence overcoming the deleterious effect of the insecticides and resulting in the evolvement of insecticide resistance [67]. Xenobiotic enzymatic detoxification occurs in two phases: phase 1 and phase 2. Phase 1 involves oxidation and reduction reactions, adding functional groups to xenobiotics while in phase 2, xenobiotics or end products of phase 1 reactions are conjugated to endogenous molecules such as glutathione [68]. Phases 1 and 2 metabolism of xenobiotics make the xenobiotics more water-soluble and easily excretable from the body [69]. Over-expression of some xenobiotics detoxifying enzymes is associated with *Anopheles*’ resistance to insecticides [70]. This over-expression can be as a result of gene amplification, changes in regulatory elements or promoter regions of genes [64]. Three main classes of xenobiotic detoxifying enzymes that contribute to insecticide resistance in *Anopheles* are cytochrome P450 monooxygenases (CYP), glutathione S-transferase (GSTs) [71] and carboxylesterases (CEs) [72].

Cytochrome P450 monooxygenases enzymes are detoxifying enzymes that participate in phase 1 of the xenobiotic metabolism. They catalyze the oxidation or reduction of compounds (endogenous and exogenous compounds) mainly into less harmful compounds by converting non-polar xenobiotics into more polar and excretable forms [73]. The end products of the reactions catalyzed by CYPs are subsequently conjugated with endogenous molecules by phase 2 enzymes, thereby making them more water-soluble and better excretable [68]. Cytochrome P450 enzymes such as CYP6M2, CYP6P1, CYP9K1, CYP6P3, CYP4H17, CYP6Z1 and CYP6Z2 have been associated with insecticide resistance in *An. gambiae* [71, 74–77], while CYP6P9a and CYP6P9b contribute to resistance in *An. funestus* [78]. In a study by Weedall et al. [79], *An. funestus* mosquitoes having a homozygous resistance allele, CYP6P9a_R were observed to have a high resistance to pyrethroid insecticides and ITNs. Their study revealed two findings: first, that a polymorphism in the cis-regulatory element drives this allele and second, that upon exposure to ITNs, mosquitoes with this allele had a greater survival and subsequently were more successful in blood-feeding than mosquitoes without this allele. Table 1 gives an overview of CYP450 and other metabolic enzymes that contribute to the development of insecticide resistance in different *Anopheles* species. Aside from contributing to insecticide resistance, CYP 450s are also involved in the bioactivation of organophosphate insecticides [80]. Many organophosphate insecticides are not active anticholinesterase, they require bioactivation by CYP 450s converting them from their phosphorothionate form to a toxic oxon form which inhibits AChE [81]. A good example of this is parathion, which is converted into paraoxon (the active acetylcholinesterase inhibitor) by CYPs [82].

**Table 1:** Classes of insecticides, their resistance mechanisms and associated proteins in *Anopheles* species

Insecticide class	Resistance mechanism and associated proteins	
	Target site	Metabolic resistance
Organophosphates	AChE-G119S in *An. gambiae*, *An. arabiensis*, *An. coluzzii*, *An. albimanus* [106–108, 253, 254]	GSTE2 in *An. gambiae* [20]
		α- and β-esterases in *An. stephensi* [99]
Organochlorines, e.g. DDT	Target site is not a metabolic protein	CYP6M2, CYP6P3, GSTD3, GSTE2, in *An. gambiae* [255]
		CYP6P9a, CYP6P9b, GSTD1-5, GSTD3, GSTE2, α-esterase in *An. funestus* [78, 256]
Pyrethroids	Target site is not a metabolic protein	CYP4G16 (cuticular resistance), CYP6M2, CYP6P3, CYP6Z2, COEAE1D, GSTE2, GSTD1, GSTD3, GSTE4 in *An. gambiae* [20, 66, 91, 95, 101, 257, 258]
		CYP6M7, CYP6P9a, CYP6P9b, CYP6P4, CYP9J11, CYP9K1 in *An. funestus* [78, 79, 256, 259, 260]
		CYP6AA3 and CYP6P7 in *An. minimus* [261]
		CYP325C1, GSTS1-1, GSTS1-2, GSTMIC2, COEJHE2, AnstABCB2, AnstABCBmember6, AnstABCG4 in *An. stephensi* [104, 262]
		CYP6P1, CYP6Z1, CYP6Z3, CYP9K1, CYP9J5, CYP9M1, GSTE2, GSTE5, GSTM1, GSTMS3, GSTS1-2, GSTS1-1, GSTE4, COEAE3G, COEAE4G, COEAE5G in *An. coluzzii* [77, 90, 100]
		CYP6AG2, CYPZ1, TPX2, CYPZ2, CYP6P1, CYP6P4, GSTE4 in *An. arabiensis* [95, 263, 264]
		CYP4H14, CYP6AA1, CYP6M3, CYP6M17, CYP6P2, α-esterase 10, AChE1 in *An. sinensis* [72, 265]
		CYP4C26, CYP6P5, CYP9K1 in *An. albimanus* [266]
Carbamates	AChE-G119S in *An. gambiae*, *An. arabiensis*, *An. coluzzii*, *An. albimanus*. N485I in *An. funestus* [106–108, 110, 254]	CYP4H17, CYP6P3, CYP6Z3, CYP6Z1, CYP12F2, CYP6M3 CYP6P4, GSTD3 in *An. gambiae* [20, 75, 267]
		CYP6M2, CYP6P3, CYP6Z1 in *An. funestus* [268]

Glutathione S-transferases (GSTs) are phase 2 detoxifying enzymes [68]. They catalyze the conjugation of endogenous compounds or xenobiotics with glutathione, thus detoxifying the xenobiotics, increasing their solubility and leading to their excretion from the mosquito [67]. GSTs are known to detoxify organophosphate insecticides, metabolize DDT to dichlorodiphenyldichloroethylene (DDE), a non-toxic metabolite [83, 84], and contribute to pyrethroid resistance [85]. Functionally, GSTs sequester the pyrethroid insecticides or detoxify their lipid peroxidation products, thereby reducing the capacity of pyrethroids to cause oxidative stress and death of the mosquitoes [86]. Over-expression of GSTs has been implicated in resistance to all the main classes of insecticides used in malaria vector control. There are about 13 classes of GSTs, with four classes, i.e. Omega, Theta, Zeta and Sigma, occurring in almost all metazoans [87] while Delta (GSTD) and Epsilon (GSTE) occur exclusively in insects and are key players in insecticide resistance [88, 89]. For example, GSTE2, GSTE5, GSTM1, GSTMS3 and GSTS1-2 have been implicated in pyrethroid resistance in *An. coluzzii* [90], while GSTD3, GSTE2 and GSTS1-2 are associated with insecticide resistance in *An. gambiae* [71, 75, 91].

Apart from the increased expression of certain GSTs, mutations in GSTs contribute to insecticide resistance. An example is a naturally occurring single amino acid mutation L119F (leucine to phenylalanine) in GSTE2, which confers resistance to DDT in *An. funestus* [92, 93]. This mutation modified GSTE2-DDT binding cavity, increasing accessibility of DDT by GSTE2 and consequently increased detoxification of DDT to DDE, thereby resulting in resistance to DDT [93]. In a study by Pontes et al. [94], I114T/F120L mutation in GSTE2 of *An. gambiae* was observed to cause structural rearrangement with a displacement of a Glu116 residue. The displaced Glu116 was postulated to act as a base that activated GSH, which bound DDT, leading to DDT resistance in the mosquito [94]. Increased expression of GSTE4 is associated with pyrethroid resistance in *An. gambiae* and *An. arabiensis*. However, rather than metabolize pyrethroids, this enzyme binds and sequesters them, thus leading to pyrethroid resistance [95]. Interestingly, vector competence, which is the capability of a vector to acquire, maintain and successfully transmit a pathogen, may be affected by the L119F GSTE2 allele [96, 97]. Ndo et al. [97] observed that the frequency of this resistant allele was significantly higher in non-infected *An. funestus* mosquitoes (55.88%) compared to their *P. falciparum* infected counterparts (40.99%). However, *An. funestus* mosquitoes with the resistant allele had higher parasite load compared to the susceptible mosquitoes. While the obtained results were contradictory, their study suggested that L119F GSTE2 may impact vector competence by increasing parasite load. A review on the effect of insecticide resistance on *Plasmodium* development has recently been published [98]. Further studies are required to fully establish the impact of metabolic resistance on vector competence in mosquitoes.

Carboxylesterases (CEs) are another class of detoxifying enzymes that act on insecticides with ester structures by hydrolyzing or sequestering them. In *Anopheles* species, overexpression of some CEs has been associated with insecticide resistance. For example, α-esterase10 and AChE1 are the main CEs that are associated with pyrethroid resistance in *An. sinensis* [72], whereas α-esterase (gb-COEAE1G) is associated with DDT resistance in *An. funestus* [93]. Also, α - and β -esterases are upregulated in malathion-resistant *An. stephensi* [99]; COEAE3G and COEAE4G are associated with pyrethroid resistance in *An. coluzzii* [90] whereas COEAE5G is constitutively expressed in permethrin resistant *An. coluzzii* [100]. Also, genome-wide association studies (GWAS) in *An. gambiae* indicated the role of COEAE1D in insecticide resistance [101].

Transporters are involved in metabolic resistance to insecticides by transporting them away from the target. For instance, ATP-binding cassette (ABC) transporters are involved in insecticide resistance by mediating their transport out of the cell [102]. Inhibition of ABC transporters in *An. stephensi* larvae was noted to increase their susceptibility to permethrin insecticide [103]. In addition, AnstABCB2, AnstABCBmember6, AnstABCG4 were upregulated in male and female adult *An. stephensi* in response to permethrin insecticide [104]. Both studies indicate that the increased expression of these transporters upon permethrin exposure is crucial for insecticide transport out of the cell and consequently insecticide resistance.

### Target site mechanism

Target site resistance refers to target site insensitivity to insecticides, reduced ability of insecticides to bind to their protein targets due to the buildup of mutations in the target proteins [105]. These mutations are usually non-silent point mutations in genes that code for the target protein [64]. Of the four classes of insecticides commonly used, only carbamates and organophosphates target a metabolic protein - AChE. A common point mutation associated with insecticide resistance in AChE is a glycine to serine mutation, G119S in *An. gambiae*, *An. coluzzii*, *An. albimanus* [106, 107] and *An. arabiensis* [108]. Owing to the new coding numbering in *An. gambiae*, codon 119 (G119S) of AChE is now referred to as codon 280 (G280S) [109]. In addition to this mutation, N485I, which is an asparagine to isoleucine mutation in the acetylcholinesterase gene, has been associated with carbamate (bendiocarb) resistance [110]. Apart from the point mutation in acetylcholinesterase which results in an insecticide resistant copy of the *ace-1* gene denoted as *ace-1*^*R*^, gene duplication of the acetylcholinesterase gene is also linked to insecticide resistance [111]. This duplication creates a permanent heterozygote allele, *ace-1*^*D*^, i.e. a susceptible (*ace-1*^*S*^) and a resistant copy (*ace-1*^*R*^) on the same chromosome [106, 112]. Also, homogeneous duplication of the *ace-1*^*R*^ gene has been reported and mosquitoes with homogenous duplication are significantly more often resistant [111].

### Cuticular resistance

Cuticular resistance to insecticides in mosquitoes occurs when cuticular proteins are remodeled to prevent or reduce uptake of insecticides [63]. This remodeling involves increasing the thickness of the cuticle, which has been associated with insecticide resistance in *Anopheles* [113]. CYP4G16 is a metabolic enzyme involved in epicuticular hydrocarbon synthesis through the oxidative decarbonylation of aldehydes to hydrocarbons [114, 115]. CYP4G16 has a markedly increased expression in insecticide-resistant strains of *An. gambiae* [116], *An*. *arabiensis* [117] and *An. coluzzii* [90]. It is involved in the remodeling of the cuticle, thereby contributing to insecticide resistance. Balabanidou et al. [114] reported that CYP4G16 contributed to insecticide resistance by remodeling the cuticle, hence it was involved in cuticular resistance mechanism rather than in metabolic resistance like other CYPs.

Apart from the metabolic proteins with known resistant mechanisms reviewed above, some other metabolic proteins are over-expressed in insecticide resistant mosquitoes compared to susceptible mosquitoes. However, their contributions to insecticide resistance and mechanisms of actions have not been validated. In a study by Isaacs et al. [75], glycine N-methyltransferase, glyceraldehyde-3-phosphate dehydrogenase and apyrase were found to be upregulated in bendiocarb resistant *An. gambiae*. Riveron et al. [93] observed that thioredoxin peroxidase (TPX2), sterol desaturase, bifunctional purine biosynthesis protein, sorbitol dehydrogenase, UDP-glucuronosyltransferase (UGT), calcium-transporting ATPase, catalase, and short-chain dehydrogenases were up-regulated in DDT resistant *An. funestus* mosquitoes. A different study on pyrethroid resistance in *An. coluzzii* revealed that chymotrypsin-1, aquaporin and ATP synthase levels were elevated in the resistant mosquitoes compared to the susceptible mosquitoes [90]. In a recent study on *An. sinensis*, upregulated expression of UGT308D3 and UGT302A3 were associated with pyrethroid resistance [118]. In *Cx. pipiens*, carbonic anhydrase, trehalase and chitin synthase were reported to contribute to pyrethroid resistance [119, 120]. In *Anopheles*, there is need to validate the possible contributions of these proteins to insecticide resistance and their mode of actions.

## Metabolic proteins, blood digestion, immune response and *Plasmodium* parasite development in *Anopheles*

The basal metabolic activities of *Anopheles* mosquitoes are sustained by feeding on sugar meals. However, female *Anopheles* mosquitoes require a blood meal to obtain the needed proteins for egg development [121]. When blood is ingested from malaria infected individuals, *Plasmodium* parasites are ingested as well by *Anopheles*. The ingested blood must be digested to release nutrients required for oogenesis. This process necessitates the activation and involvement of several metabolic proteins [122]. Heme in blood also triggers the heme detoxification pathway and the presence of parasites triggers the immune response in the mosquito [123]. In all these processes, metabolic proteins play crucial roles in contributing to blood digestion, parasite development or removal, and consequently malaria transmission or prevention.

### Metabolic proteins and blood digestion in *Anopheles*

Blood digestion in *Anopheles* is a well-coordinated process and studies involving transcriptomic and proteomics analyses comparing sugar-fed and blood-fed mosquitoes revealed that metabolic proteins are critical in blood digestion [124, 125]. The ingested blood is transported to the midgut and induces the synthesis of the peritrophic membrane [124]. Formation of the peritrophic membrane is important because iron from heme of the blood and human antibodies can harm the mosquitoes [125]. Thus, this membrane protects the mosquitoes. The peritrophic membrane surrounds the blood meal, regulates the digestion rate by controlling the translocation of digestive enzymes and digestion products across the membrane [126]. It also regulates heme detoxification and provides a physical barrier, which is the first level of defense against *Plasmodium* parasites [123, 127]. In the midgut, proteases and other digestive enzymes break down the ingested blood, and the resulting nutrients are processed in the fat body and taken up by the ovaries for egg development [128].

Blood meals have a large content of proteins [122, 129]. Thus, proteolytic proteins involved in protein digestion are highly expressed in blood-fed mosquitoes and catalyze the cleavage of proteins into amino acids. This is important because seven amino acids (leucine, valine, isoleucine, phenylalanine, lysine, arginine and histidine) are essential for egg development in mosquitoes. Hence, they must be obtained from blood meals [130]. Several transcriptome studies in *An. stephensi* and *An. gambiae* revealed that proteases such as trypsin 1 and 2, chymotrypsin, carboxypeptidase, aminopeptidase and a serine protease were highly expressed in blood-fed females when compared with their sugar-fed counterparts [128, 131–133]. Interestingly, these proteases have been reported to contribute to *Plasmodium* clearance in the mosquito. For example, a proteomic study that compared species of *An. culicifacies* mosquitoes that are susceptible to *Plasmodium* infection with their refractory counterparts, revealed that chymotrypsin 2 was upregulated in the refractory species and that chymotrypsin 2 may be involved in preventing *Plasmodium* development in the mosquitoes [134]. This may be explained by the destruction of ookinetes by the proteolytic enzyme since early forms of *Plasmodium* parasite in the mosquito, within 24 h post-blood-feeding (pbf), are vulnerable to the action of digestive enzymes [135, 136]. These parasite forms include gametocytes, zygotes and undifferentiated ookinetes. Baton & Ranford-Cartwright [136] compared the time points at which peak expression levels of trypsin and chymotrypsin occurred in *An. albimanus* and *An. stephensi*. They observed a peak expression at 14 h and 20 h in *An. albimanus* as opposed to 30 h and 36 h in *An. stephensi* for the two enzymes trypsin and chymotrypsin, respectively. This difference may contribute to the disparity in *Plasmodium* susceptibility in the two mosquito species. While *An. albimanus* was refractory to *P. falciparum* (3D7A), *An. stephensi* was susceptible to it [136]. Their study revealed that early expression of digestive enzymes following a blood meal may be important for parasite clearance, and that the time at which peak expression of proteases (and other digestive enzymes in extension) is achieved, differed across *Anopheles* species. In addition, this difference in peak expression time may explain the downregulated levels of trypsin that was observed at 24 h pbf in other studies, e.g. in *An. dirus* [125]. Therefore, in studying expression patterns of proteases and metabolic proteins in general, and their impact on *Plasmodium* development in *Anopheles* species, a time series experiment may be more revealing.

Proteases act on parasite forms that are close to the peritrophic membrane. However, parasites that are farther away in the center of the blood meal are able to gain time and differentiate into mature forms, capable of responding to and escaping from the action of the digestive enzymes [137]. In a study by Baia-da-Silva et al. [135], the development of peritrophic membrane in *An. aquasalis* was hindered and the effect of this absence on *Plasmodium vivax* development was verified. They observed that the absence of this membrane enhanced interaction of digestive enzymes with parasites and resulted in increased parasite killing. They reported that trypsin contributed to parasite clearance in mosquitoes lacking peritrophic membrane and the subsequent treatment with a trypsin inhibitor increased infection intensity [135]. However, an earlier study by Shahabuddin et al. [138], showed that *Plasmodium* responded to elevated trypsin-like protease levels by increased secretion of chitinase, with which it digested the peritrophic membrane and avoided the action of digestive proteases. Huber et al. [139] reported that parasite chitinase was not secreted until about 15–20 h pbf of mosquitoes when the parasites were developing from zygotes to ookinetes. Also, they suggested that the effect of proteases on parasite development depended on the timing of protease expression and the level of interactions or contact of digestive proteases with parasites [139]. A study that compared *An. dirus* strains susceptible to *P. yoelii nigeriensis* with refractory strains showed that trypsin and aminopeptidase expression were not different between the two strains [140]. A similar study that compared *An. stephensi* strains susceptible to *P. falciparum* with refractory strains revealed that trypsin activity was not different between the two strains, though, aminopeptidase activity was higher in refractory mosquitoes [141]. While these two studies suggest that trypsin does not affect *Plasmodium* development, other studies showed that trypsin affects *Plasmodium* development in other *Anopheles* species. These studies suggest that increased early expression of trypsin and chymotrypsin pbf contributes to parasite clearance in mosquitoes. Thus, strategies that can reprogram mosquitoes to prevent peritrophic membrane development or to express proteases early enough upon blood-feeding (i.e. before parasites differentiate into forms that can respond to protease activity by secreting chitinase), may decrease parasite development in the mosquitoes thereby preventing malaria transmission. However, the possibility and sustainability of this reprogramming remains a question to be answered.

Shi et al. [142] assessed the expression levels of carboxypeptidase A and B in *An. sinensis* (AsCPA and AsCPB) 24 h pbf and noted that five out of the eight carboxypeptidases that were present in the mosquito were upregulated upon blood-feeding, i.e. AsCPA-I, AsCPA-III, AsCPA-IV, AsCPA-VI and AsCPB-II. This may point to their probable role in blood digestion. Similarly, carboxypeptidase (CPA) levels were observed to be significantly elevated in *P. berghei* infected, blood-fed *An. stephensi* mosquitoes compared to non-infected, blood-fed controls [143]. In their study, feeding mosquitoes with *P. berghei* parasitized blood meal containing CPA targeting antibodies, hampered the development of the parasite in the mosquito’s midgut [143]. Furthermore, their study revealed the importance of carboxypeptidase in *Plasmodium* parasite development in mosquitoes. Thus, carboxypeptidase can be inhibited or targeted with antibodies to prevent malaria transmission.

Carbohydrates are important energy sources in insects that can be obtained from their diet directly or synthesized from amino acids or lipids. As such, enzymes involved in carbohydrate and lipid metabolism such as lipases, adenosine monophosphate (AMP) dependent ligase, α-glucosidases and α-amylases are differentially expressed during a blood meal [132, 134, 144]. In addition, the pentose phosphate pathway is associated with blood digestion [145]. Metabolomics analysis of *An. gambiae* 24 h pbf by Champion & Xu [146], revealed an increased concentration of glucose 6 phosphate and 6-phosphogluconate. This may be indicative of increased expression of the enzymes that are involved in their production, namely, glucose-6-phosphate dehydrogenase (G6PDH) and 6-phosphogluconate dehydrogenase (6-PGDH). These reactions were necessary for replenishing NADPH levels, a metabolite that is needed for maintaining the redox metabolism [145]. This replenishment is very important because reactive oxygen species (ROS) generation is increased during blood-feeding, particularly during parasitemia [147]. In female mosquitoes, nutrients from digested blood are transported to the ovary for oogenesis. This requires the action of transporters such as lipid transporters that mobilize lipids from the midgut to the ovaries [148]. Lipids are generally important components of cell membranes and include fatty acids, phospholipids and sterols. Four lipid transporters have been observed to be upregulated pbf in *Anopheles* [149].

After a blood meal, *Anopheles* mosquitoes reduce their flight activity and seek a resting place. In blood-fed *An. gambiae* mosquitoes, pyrroline-5-carboxylate reductase and proline oxidase were reported to be downregulated. Both enzymes are required for the metabolism of proline for energy production during flight [144]. Inhibition of any of these enzymes can prevent flight of mosquitoes, thereby limiting their subsequent access to humans for malaria transmission. Although there is currently no evidence for the use of these kinds of inhibitors in vector control, evidence exists that the inhibitors of enzymes involved in energy production during flight can reduce flight activity. Generally, insects differ in the substrate used to fuel flight, varying from the use of trehalose to the use of diacylglycerol or proline [150]. For example, while blood-sucking insects like *Anopheles*, *Aedes* and tse-tse fly use proline, some other insects, such as locust and, cockroaches use trehalose instead [151–154]. In *Aedes*, a combination of proline and pyruvate (pyruvate can be obtained from trehalose metabolism since trehalose is the major sugar in insect hemolymph) provided the highest energy needed for flight [155]. Similarly, *Anopheles* can use proline and pyruvate to fuel flight [154]. Exposure of cockroaches to a trehalase inhibitor, validoxylamine A, thus preventing trehalose metabolism, led to a 70% reduction in flight muscle activity and prevented the cockroaches from flying for > 2.5 min compared to active (1–5 min) and very active (> 5 min) controls [152]. Similarly, validamycin A, a trehalase inhibitor, prevented flight in adult *Ae*. *aegypti* mosquitoes in a dose-dependent manner. Mosquitoes exposed to 0.5 mg/ml of validamycin A were unable to fly at all [156]. In addition, validamycin A decreased egg hatching, pupation time and prevented emergence of female *Ae*. *aegypti* mosquitoes, thus, offering multiple control strategies [156]. Gleaning on these and considering the fact that the ability of insects to fly is crucial to seek a host for transmission, inhibiting pyrroline-5-carboxylate reductase, proline oxidase or trehalase needed to provide fuel for flight may help reduce malaria transmission. Inhibitors can be designed for these targets, which can be incorporated into insecticides.

Ingestion and digestion of a blood meal in mosquitoes lead to the production of reactive species such as hydrogen peroxide (H_2_O_2_), so causing oxidative stress in the mosquito [157]. Some antioxidant enzymes such as catalase, help to scavenge these free radicals, thereby reducing oxidative stress and preventing subsequent damage to the mosquitoes. Catalase is an antioxidant enzyme that breaks down H_2_O_2_, thereby preventing the formation of the hydroxyl radical. The levels of H_2_O_2_ in the hemolymph are significantly higher in *An. gambiae* strains that are refractory to *Plasmodium* compared to the susceptible strains [157]. dsRNA silencing of catalase resulted in reduced ookinete survival in *An. gambiae* G3 strains [158]. In another study, silencing of catalase in *An. gambiae* and subsequent blood-feeding of mosquitoes resulted in higher mortality [159]. Therefore, catalase plays a crucial role in regulating immune response to *Plasmodium* parasite and ensuring the survival of the mosquitoes. Inhibiting catalase offers multiple ways of preventing malaria transmission, by resulting in the death of the mosquito or by supporting parasite clearance in the mosquito.

### Metabolic proteins and immune response to *Plasmodium* infection in *Anopheles*

When *Plasmodium* is picked up during ingestion of parasitized blood in mosquitoes, the innate immune system of the mosquito is triggered and tries to eliminate the intruding parasite [160]. The first level of defense is the physical barrier, i.e. the peritrophic membrane. *Plasmodium* parasites that successfully emerge from the peritrophic membrane, encounter another level of defense known as the innate immune responses in the mosquito involving processes such as phagocytosis, melanization and lysis [2, 4]. These processes result in massive parasite losses and only parasites that escape this immune response develop into sporozoites that can be transmitted during a subsequent blood meal [4]. The immune response process in *Anopheles* is the subject of several reviews [161–164]. The process involves the action of some metabolic proteins that are discussed in this subsection.

Difference in metabolic activities between refractory and susceptible strains of *An. gambiae* have been reported to influence their susceptibility to *P. berghei* infection [165]. These differences include increased expression of glycolytic enzymes and impaired mitochondrial respiration leading to increased generation of ROS in refractory strain (*An. gambiae* L3-5 strain) compared to the susceptible strain (*An. gambiae* G3 strain) [165]. In addition, the increased ROS generation resulted in higher parasite clearance through melanization but with fitness costs because the refractory strains had a lower lifespan than the susceptible strains due to the damaging effects of ROS [165].

ROS mediates *Anopheles* immunity [158, 166]. The increased generation of these reactive species such as superoxide anion, H_2_O_2_, nitric oxide (NO) in *Anopheles*, limit the development of *Plasmodium* in the mosquito [167, 168]. Heme peroxidase, HPX2 and NADPH oxidase 5 (NOX5) in *An. gambiae* were involved in *P. berghei* clearance through nitration of epithelial cells [169]. Also, increased expression of nitric oxide synthase (NOS), the enzyme that synthesizes NO, and enhanced peroxidase activity are important steps in the *Anopheles* immune response to *Plasmodium* infection [170]. NOS, NOX5 and HPX2 are important for *Anopheles* immune response to parasite challenge since they mediate epithelial nitration, marking the parasite for clearance by TEP-mediated lysis [169]. NO activates the synthesis of antimicrobial peptides (AMP) that are responsible for parasite killing [171]. Luckhart et al. [172] reported that inducible NOS were upregulated in *An. stephensi* upon infection with *Plasmodium* parasite. Also, they noted that inhibiting NOS reduced parasite clearance while providing L-arginine (a substrate required by the enzyme for the synthesis of nitric oxide), enhanced parasite clearance [172]. Kajla et al. [173] discovered that heme peroxidase 15 (HPX 15) suppressed immune response of *An. stephensi* to *Plasmodium* infection by preventing the recognition of the parasite. They found that silencing HPX15 resulted in increased expression of NOS and parasite clearance [173]. These studies suggest that modulation of inducible NOS levels or activity can enhance refractoriness of mosquitoes to parasite thus preventing malaria transmission.

Clip domain serine protease (CLIP) could positively or negatively regulate TEP-mediated killing of *Plasmodium* parasite as well as take part in the melanization process of the immune response to *Plasmodium* infection [174]. Nakhleh et al. [174] found CLIPA14 to negatively regulate mosquito’s immune response because its knockdown resulted in increased melanization of *Plasmodium* parasites. Similarly, a different study identified CLIPA2, CLIPA5 and CLIPA7 as negative modulators of immunity [175]. CLIP serine proteases with a positive modulating effect on mosquito immune response, e.g. CLIPA8, proteolytically activate prophenoloxidase (PPO) to phenoloxidase (PO) [2]. PO catalyzes the biosynthesis of reactive quinines from tyrosine and 3,4-dihydroxyphenylalanine [176]. The resultant quinines produce melanin that crosslinks proteins and forms a capsule around the parasite during encapsulation response against *Plasmodium* and other parasites [175].

### Role of metabolic proteins in *Plasmodium* parasite development

Although not directly involved in *Anopheles* immune response to *Plasmodium*, some metabolic proteins have been reported to either aid or suppress the development of *Plasmodium* in mosquito and could serve as possible targets for the prevention of malaria transmission. Examples of these proteins are aquaporin 3 (AgAQP3), trehalose transporter (AgTreT1) and kynurenine 3-monooxygenase (AgKMO) [177–179].

AgAQP3 transports water, glycerol and urea. It is important for the survival of *Anopheles* and development of *Plasmodium* parasite in the mosquito [178]. Knockdown of AgAQP3 using RNAi, reduced median survival of *An. gambiae* at 39 °C and resulted in decreased *Plasmodium* oocytes formation in the midgut of *Anopheles*, indicating decreased vector competence [178]. The observed effect of AgAQP3 knockdown was attributed to the importance of AgAQP3 in controlling post-prandial diuresis and maintaining the osmotic balance in the mosquito. Accumulation of glycerol by aquaporin in the cell is also required by the mosquito to enhance its tolerance to cold [180]. Therefore, AgAQP3 is important for both parasite transmission and mosquito survival and may serve as an insecticide target or a target for disruption of parasite development. Target-specific inhibitors of this protein can be designed as insecticides or transmission-blocking agents.

AgTreT1, a trehalose transporter that transports trehalose from the fat body to the hemolymph [177] was observed to be a positive modulator of *Plasmodium* in *Anopheles.* Trehalose is an important sugar in insects that helps in regulating the temperature of the insects, thus preventing them from the lethal effects of cold. Silencing of the trehalose transporter using RNAi increased *Anopheles* refractoriness to *Plasmodium* [177]. Since aquaporin and trehalose contribute to the maintenance of warm temperature in *Anopheles*, a condition needed for *Plasmodium* development and survival of *Anopheles*, these proteins could serve as possible targets for malaria vector control.

Two main nutrient transporters, lipophorin and vitellogenin, produced by the fat body influence *Plasmodium* development in *Anopheles* [5]. Lipophorin is a diacylglycerol-carrying lipoprotein, necessary for transporting lipids while vitellogenin is a protein precursor of egg yolk. These two proteins reduce the parasite-killing potential of TEP1, a major protein involved in the lysis of *Plasmodium* parasites during *Anopheles*’ immune response upon exposure to the parasite [5]. This makes *Anopheles* more susceptible to *Plasmodium*, consequently making it capable of transmitting malaria. Inhibiting these proteins may increase TEP1-mediated lysis, thereby making the mosquito more refractory to *Plasmodium*. However, inhibiting these proteins would negatively impact egg development. In contrast, while lipophorin and vitellogenin were upregulated in response to blood-feeding, another lipid transporter, apolipophorin (ApoLp) was downregulated in blood-fed mosquitoes [130]. Kamareddine et al. [181] reported that apolipophorin was a negative regulator of thioester protein (TEP) induced immune response. They noted that silencing the apolipophorin gene using RNA interference (RNAi), led to increased TEP expression [181]. Similarly, silencing of ApoLp-III in *An. stephensi* led to enhanced induction of NOS, which is important for *Plasmodium* clearance [182]. Therefore, strategies to downregulate ApoLp in mosquitoes upon blood-feeding may be essential for increased expression of NOS and effective TEP lysis of *Plasmodium* parasite.

Kynurenine 3-monooxygenase (KMO) is a key enzyme in the biosynthetic pathway that produces xanthurenic acid (XA), which is required to activate guanylyl cyclase [183, 184]. KMO catalyzes the conversion of L-kynurenine to 3-hydroxy-L-kynurenine, which is processed in a subsequent reaction to XA [185]. XA has been identified as a gamete-activating factor of *Plasmodium* [184]. The activation of guanylyl cyclase by XA is important for the completion of *P. berghei* development in the midgut of mosquitoes [186]. Knockout of the *KMO* gene in *An. stephensi* using transcription activator-like effector nucleases (TALEN) resulted in XA-deficient mosquitoes that had reduced oocytes and sporozoites in their midgut and salivary gland, respectively [179]. Also, the study highlighted the important effects of xanthurenic acid on the development of *Plasmodium* in *Anopheles* mosquito and suggested that KMO is a possible target for blocking malaria transmission. 3-hydroxy-L-kynurenine produced by KMO in the XA biosynthetic pathway is further metabolized by 3-hydroxykynurenine transaminase (3HKT). 3HKT metabolizes 3-hydroxy-L-kynurenine to XA, thus preventing the accumulation of potentially toxic 3-hydroxy-L-kynurenine [187]. 3HKT of *An. gambiae* has been cloned, expressed, purified, and its biochemical activity and the 3-dimensional (3D) structure determined [188, 189]. Its inhibition can hamper *Plasmodium* replication in mosquitoes, since its inhibition prevents XA synthesis needed to trigger exflagellation and maturation of the *Plasmodium* male gametes [190].

While 3HKT and KMO could serve as a possible target for malaria transmission-blocking strategies by preventing parasite development in the mosquito, inhibiting these enzymes can negatively affect survival of the mosquitoes. The inhibition of 3HKT results in the accumulation of 3-hydroxy-L-kynurenine, which could undergo rapid oxidation to form free radicals that can induce apoptosis [189]. Thus, inhibitors of 3HKT may act as both potential insecticides and transmission-blocking agents. 3HKT has been observed to be the target for 1,2,4-oxadiazole compounds having larvicidal activity against *Ae*. *aegypti* [191, 192]. Since 3HKT of *An. gambiae* shares 43% sequence similarity with 3HKT of *Ae*. *aegypti* [188], 1,2,4-oxadiazole compounds could be starting compounds for identification of novel insecticides or transmission-blocking agents. Inhibition of KMO prevents synthesis of 3-hydroxy-L-kynurenine, which is essential for the development of compound eye in mosquito pupa stages [185]. Consequently, knockout of this gene will result in mosquito mutants with impaired eye development, since 3-hydroxy-L-kynurenine cannot be produced during the larvae stage. Reports of impaired eye development due to KMO knockout have been reported in *Ae*. *aegypti*, with generated mutants having white eye phenotypes [193, 194]. Since compound eye development is completed at the adult stage of mosquitoes, chemical inhibitors of KMO could serve as possible transmission-blocking agents without affecting eye development.

### Role of metabolic proteins in fecundity

Fecundity is a measure of the number of eggs or offspring an organism can produce. This is dependent on egg maturation in the ovaries and oviposition (egg-laying) [195]. Some metabolic proteins (enzymes and transporters) involved in blood digestion and metabolism are upregulated in blood-fed mosquitoes and affect their fecundity [130, 149, 196, 197].

One of such enzymes is phenylalanine-4-hydroxylase (PAH), an enzyme involved in amino acid metabolism, which converts phenylalanine to tyrosine. One isoform of PAH was found to be 3.2-fold over-expressed in blood-fed *An. stephensi* [130]. This enzyme is important for survival and fecundity in insects [198]. Fuchs et al. [196] reported that knockdown of PAH in *An. gambiae* reduced the number of eggs laid by the mosquitoes, and impaired the melanization of *Plasmodium berghei* ookinetes and mosquito eggs. These observations were linked to the unavailability of tyrosine for further metabolism to yield dopamine and melanin after silencing PAH. This was further confirmed by inhibiting another enzyme in the dopamine and melanin synthesis pathway, DOPA decarboxylase (DDC) with Carbidopa. DDC catalyzes the formation of dopamine. Inhibition of DDC yielded the same phenotypes as with PAH silencing [196]. Their study affirms the importance of phenylalanine and tyrosine metabolism in the fecundity of mosquitoes and their immune response to *Plasmodium*. While PAH might be a potential target for sterilizing strategies through its inhibition, its inhibition will hamper melanization of parasites, consequently, this might increase parasite transmission. This nullifies PAH inhibition as a strategy for vector control. On the other hand, generating mosquitoes’ strains with enhanced expression of PAH pbf, might result in mosquitoes with higher fecundity (more eggs) and enhanced immune response (increased melanization). This strategy is promising, if successful, as the refractory mosquitoes would pass on this mutation to their offspring and these mutants will possibly compete well with wild type mosquitoes in nature.

Another enzyme involved in amino acid metabolism, ornithine decarboxylase is encoded by three genes that are upregulated pbf [149]. Ornithine decarboxylase catalyzes the decarboxylation of ornithine to form putrescine [199]. Also, it is important for cell growth because it catalyzes the committed step in polyamines production required for stabilizing newly synthesized DNA [199]. This enzyme is important in fecundity as DNA synthesis and cell cycle are integral processes that accompany egg development and embryogenesis. Inhibition of ornithine decarboxylase using α-difluoromethylornithine in *Ae*. *aegypti* resulted in reduced vitellogenin levels, thus negatively affecting fecundity [197]. In addition, increased ornithine decarboxylase pbf led to sequestering of arginine for polyamine synthesis, making it unavailable for nitric oxide synthesis which is needed for immune response to *Plasmodium* parasites [200]. Thus, inhibiting ornithine decarboxylase would both provide sterilizing strategies as well as result in increased nitric oxide expression for immune response and parasite clearance.

Heme oxygenase, which catalyzes the degradation of heme, also plays a role in fecundity of mosquitoes. Heme is an important component of human blood, which is highly toxic to mosquitoes [201, 202]. Heme oxygenase catalyzes the degradation of heme; thus, it is important for protecting the mosquito from heme toxicity. Approximately 13% of the heme contained in ingested blood is incorporated into the mosquito as follows: 7% into tissues of the adult mosquito and 6% into its eggs [203]. Spencer et al. [6] reported that the consumption of heme oxygenase inhibitors such as zinc protoporphyrin (ZnPP) and tin protoporphyrin (SnPP) by *An. gambiae* remarkably decreased egg-laying. They noted that inhibition of heme oxygenase increased sterility by preventing oviposition (laying of eggs), consequently culminating in reduced availability of vectors for malaria transmission [6]. So, the inhibition of heme oxygenase may be further studied for its sterilizing effect for mosquito population suppression.

Similarly, catalase plays a crucial role in regulating fecundity. dsRNA-mediated knockdown of catalase has been observed to significantly reduce the fecundity of *An. gambiae* mosquitoes [204]. Therefore, catalase may serve as a target for sterilizing strategies. Meanwhile, an 80% reduction in egg hatching was observed in sterol deficient female houseflies suggesting that sterols are essential for egg hatching [205]. Therefore, transport and metabolism of lipid from a blood diet is crucial in *Anopheles* reproduction. Vitellogenin is a precursor protein for egg yolk formation, belongs to a family of proteins involved in lipid transport. It is elevated in blood-fed mosquitoes compared to the sugar-fed controls [130]. Since vitellogenin also plays a role in downregulating the anti-plasmodial response in the mosquitoes, it could be inhibited to provide both sterilizing and transmission-blocking strategies.

## Metabolic proteins and vector control using insecticides: the way forward

To combat the increasing insecticide resistance in mosquitoes, development of new insecticide molecules and combinatorial strategies can be adopted. Identifying novel insecticide targets or taking advantage of unique features in known insecticide targets can help the development of highly selective insecticides. Furthermore, combinatorial strategies may be followed by combining an insecticide with another, with synergists, with sterilants or with transmission-blocking agents in order to slow down resistance and also provide multiple ways of controlling the vector.

### Identifying novel insecticide targets

Following the path to identifying metabolic targets that are (i) crucial for survival in *Anopheles* species, and (ii) share little or no similarity with other non-target species, developing suitable inhibitors for them will provide a wide array of molecules to replace the classes of insecticides currently being used in malaria control. Previous studies have suggested some metabolic proteins as potential insecticide targets such as carbonic anhydrases, arylalkylamine N-acetyltransferases, V-ATPase, PFK, chorion peroxidase and seven computationally predicted potential insecticide targets [49–52, 206, 207].

Carbonic anhydrases (CAs: EC 4.2.1.1), the enzyme that catalyzes the reversible hydration of carbon dioxide to bicarbonate, has several classes such as α-, β-, γ-, δ-, ζ-, η- and ɵ-CAs [208, 209]. Aside from genes that encode α-CAs in *An. gambiae*, Vullo et al. [52] identified a gene that encodes a β-CA in *An. gambiae*, which is absent in vertebrates [210]. In their study, series of anion inhibitors were tested against this metabolic target. Sulphamide, sulphamic acid, phenylboronic acid and phenylarsonic acid successfully inhibited carbonic anhydrase. Although the inhibitors tested were not specific for β-CAs, their study revealed that specific inhibitors of β-CAs can be used selectively against invertebrates with minimal toxicity to vertebrates. Recently, famotidine, an antiulcer drug was successfully used to inhibit *Anopheles* β-CA with inhibition constant of 397 nM [211]. This particular study is noteworthy since famotidine is safe for humans. Thus, specific *Anopheles* β-CA inhibitors that have no toxicity in humans might serve as novel insecticides for malaria control. However, it must be determined if β-CA is indeed essential for the survival of *Anopheles* since there are α-CAs present that may confer redundancy.

Arylalkylamine N-acetyltransferases (aaNAT, EC 2.3.1.87) catalyze the acetylation of arylalkylamine such as acetylation of dopamine to N-acetyldopamine [212]. These enzymes are necessary in neurotransmitter metabolism and insect cuticle sclerotization [213]. Unlike humans that have one aaNAT, insects have multiple aaNAT, one of which is dopamine N-acetyltransferase (DAT) that is conserved in all insects. However, some aaNAT are insect specific (iaaNAT) and specific for certain genera and substrates, hence they can serve as possible targets for more specific insecticides [214]. O’Flynn et al. [51] revealed that residues that make up the amine binding pocket and the CoA binding pocket of iaaNAT varied among different genera. Their study suggests that these genera specific residues could be exploited to create genus specific insecticides. In previous studies, knockdown of iaaNAT in *Bombyx mori* and *Tribolium castaneum* resulted in increased melanin deposition and compromised structural integrity of the exoskeleton [215, 216]. These changes could affect the ability of the insects to mate as well as make them more susceptible to damages from environmental threats [51]. While compromise of structural integrity was recorded in these studies, no direct mortality resulting from inhibition of iaaNAT was reported. Further studies are required to characterize iaaNAT in *Anopheles*, elucidate the effects of their inhibition and the possibility of these iaaNATs serving as potential insecticide targets.

V-ATPases are proton pumps that hydrolyze ATP and use the energy obtained from the hydrolysis to transport protons across membranes, thus maintaining the intracellular and extracellular pH of cells [217]. Two insecticidal molecules isolated from plants have been observed to inhibit insect V-ATPases: (i) dihydroagarofuran sesquiterpene polyesters isolates obtained from the root bark of Chinese bittersweet (*Celastrus angulatus* Max) inhibit subunit H of V-ATPases [49] (specifically, two of these polyesters, CV-6-α-aminopropanoicacid ester and NW70 were highly toxic to *Mythimna separata* larva with a reported LD_50_ of 33.605 and 86.271 µg/g); (ii) a peptide isolate from pea seeds (*Pisum sativum*), pea albumin 1 subunit b (PA1b) has also been observed to selectively inhibit insect V-ATPases by binding to their c and e subunits, so, PA1b was proposed to be a potential insecticide [217]. In a study by Gressent et al. [218], 250 µg/ml of PA1b was added to *Cx. pipiens* L3 larvae in water, 100% of the larvae survived after one day and 0% survival was observed after two days. Also, *Ae*. *aegypti* has been reported to be highly sensitive to PA1b [219]. Although both studies were not specific for *Anopheles*, they are pointers to the possibility of exploiting V-ATPases for vector control strategies. Studies testing these inhibitors in *Anopheles* and identifying other suitable selective inhibitors of *An. gambiae* V-ATPases are needed to fully explore and ascertain the possibility of V-ATPases serving as potential insecticide targets. Also, the safety of these molecules to humans must be extensively verified.

Phosphofructokinase (PFK, EC 2.7.1.11), a key regulatory enzyme, catalyzing the committed step in the glycolytic pathway has been proposed as a potential insecticide target [50]. In the experiments carried out by Nunes et al. [50], it was observed that PFK inhibition by ATP in *Ae*. *aegypti* was not enhanced by citrate, and AMP could not relieve ATP inhibition of PFK. Subsequent alignment of several insect PFKs and comparison with non-insect PFKs revealed that PFK in insects including disease vectors *Aedes*, *Anopheles* and *Culex*, have modified citrate and AMP binding sites that distinguish them from their orthologs in non-insect species. Amino acid residues Lys557, Lys617 (a positively charged amino acid) and Thr618 (a neutral amino acid) in citrate binding site of human PFK are substituted by Arg (a positively charged amino acid), Ser or Ala (a neutral amino acid), and Asp or Glu (a negatively charged amino acid), respectively, in all insect sequences examined in their study. In addition, amino acid substitution in AMP binding sites resulted in changes in the overall electrostatic charges of insect PFKs compared to that of humans. These substitutions in AMP and citrate binding sites make PFK in these insects insensitive to regulation by citrate and AMP [50]. Considering the importance of PFK for energy metabolism and consequent survival, these insect unique modifications in PFK could be exploited in disease vectors to produce highly specific and selective insecticides. Further studies evaluating the impact of knockdown of PFK in *Anopheles*, determining its 3D structure and identifying specific inhibitors are needed.

Computational studies have also helped in the identification of insecticide targets. For instance, Adebiyi et al. [206] employed computational techniques to predict essential metabolic reactions in *An. gambiae* (consequently, metabolic enzymes) i.e. proteins that are vital for the survival of *An. gambiae*. Of the 61 enzymes predicted as essential, seven had no homology with humans, tilapia and chicken. Specific inhibitors of these enzymes could serve as novel insecticides, so, future studies can be done to identify suitable inhibitors for these targets. However, one major limitation of the study was the exclusion of transporters that are involved in metabolism, which could be possible insecticide targets. Further computational studies can aid prediction of other potential insecticide targets that can then be confirmed experimentally. Yousafi et al. [207] using computer-aided drug design (CADD) approach to identify alternative insecticides, predicted lead molecules that selectively inhibited insect chorion peroxidase. Their study identified ZINC04581496 and ZINC15675298 as effective lead compounds for chorion peroxidase in *Ae*. *aegypti* and *An*. *gambiae*, respectively. Although these two compounds were reportedly harmless to humans since they target insect chorion peroxidase, laboratory experiments validating the effect of inhibition of chorion peroxidase on survival in mosquitoes and the suitability of the predicted lead molecules as insecticides are needed.

All the above studies are indicative of the importance of metabolic proteins as insecticide targets. For all the proposed insecticide targets, the development of novel insecticidal molecules can be guided by studies involving (i) knockdown of these proteins and evaluating their effects on *Anopheles* survival; (ii) comparing protein sequence and structure to determine organism specificity and identifying unique features in targets that can be manipulated; and (iii) 3D structural elucidation of confirmed potential targets, virtual screening and identification of lead compounds.

### Generation of organism-target specific and selective insecticides

**Fig. 4:**
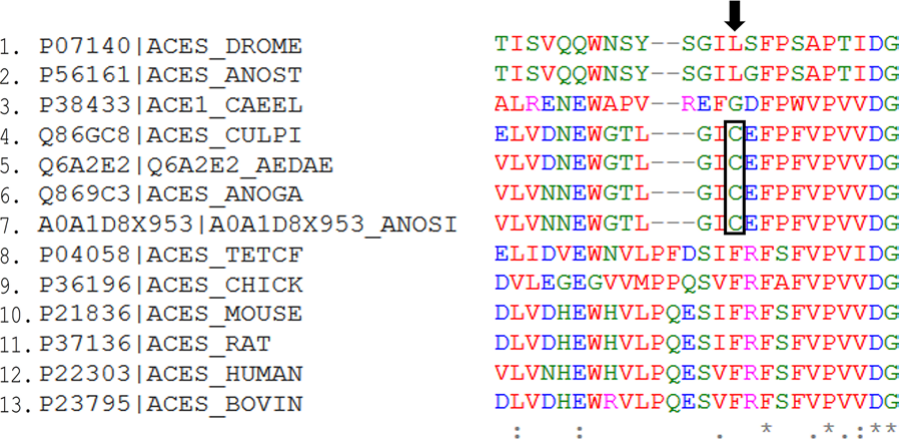
Conserved unpaired cysteine residue in the acetylcholinesterase (AChE) of disease vectors for selective insecticide design. The sequences shown are from *Drosophila melanogaster* (DROME), *Tetronarce californica* (TETCF), *Mus musculus* (MOUSE), *Homo sapiens* (HUMAN), *Bos taurus* (BOVIN), *Rattus norvegicus* (RAT), *Caenorhabditis elegans* (CAEEL), *Anopheles stephensi* (ANOST), *An. gambiae* (ANOGA), *Culex pipiens* (CULPI), *An. sinensis* (ANOSI), *Aedes aegypti* (AEDAE). The name of each organism starts with its UniProt accession number. The black arrow points to the position of the conserved unpaired cysteine residue. The unpaired cysteine residue is conserved in disease vectors (4–7). This residue is substituted by a leucine residue in *An. stephensi* and *Drosophila* AChE (1–2), phenylalanine residues in mammals, fish and bird AChE (8–13), and a glycine residue in nematode AChE (3). This unpaired cysteine could be targeted for the development of more selective and specific insecticides. * indicates positions that have single and conserved amino acid residues; : indicates conservation between amino acid residues of strongly similar properties; . indicates conservation between amino acid residues of weakly similar properties

Generation of organism-target specific and selective insecticides involves taking advantage of unique features in insecticide targets. This is highly dependent on the structural elucidation of insecticide targets and creation of more target specific inhibitors. This is important for both newly identified and already known insecticide targets, thus providing insecticides that are less toxic to non-target species. For example, structural elucidation of AgAChE revealed an unpaired cysteine (Cys), Cys447, which is absent in hAChE [25]. However, protein sequences of 13 animal species were analyzed for the conservation of this unpaired cysteine residue using Clustal Omega on EMBL-EBI’s multiple sequence alignment platform. The result showed that this cysteine residue is conserved in some of the disease vectors, namely, *An*. *gambiae*, *An*. *sinensis*, *Ae*. *aegypti* and *Cx*. *pipiens*, but absent in non-target species such as humans and birds that have other amino acid residues substituted at this position instead (Fig. 4). Although this residue is not conserved in *An*. *stephensi*, it could be utilized for selective targeting of *An*. *gambiae* and other disease vectors. A recent study tested some selected AChE cysteine-targeted insecticides (succinimide or maleimide compounds) on AgAChE and hAChE and found that all the tested compounds inhibited both enzymes irreversibly, showing poor selectivity [220]. Although the study did not support the concept of selectively targeting AgAChE by taking advantage of the unique cysteine residue, some older studies supported the cysteine-targeted selective inhibition of AgAChE. For example, Pang et al. [221] noted that 6 μM of a methanethiosulfonate-containing molecule had 95% inhibition on AgAChE and > 80% on *Ae. aegypti* and *Cx*. *pipiens* in approximately 30 minutes, while it partially inhibited hAChE after a prolonged exposure of 4 hours [221]. While their study suggests that rapid selective inhibition of AgAChE is possible, the potential hazards associated with prolonged exposure to this molecule is questionable. Dou et al. [222] observed that two maleimide compounds, PMn and PYn selectively and irreversibly inhibited AgAChE but spared that of humans. These studies indicate that species-specific or unique features in insecticide targets could be manipulated for targeted vector control. However, the concept of selective cysteine-targeted inhibition should be further studied and explored to enable the design of new compounds that will selectively and specifically inhibit AgAChE with no toxicity to non-target species especially humans.

Asides exploiting unique features in targets for insecticide design, understanding the mechanism by which mutations in current insecticide targets reduce their binding affinity for the corresponding insecticide is needed. This may give insight into ways by which current insecticides can be chemically modified to overcome insensitivity to these targets. For instance, new carbamate derivatives synthesized *via* chemical substitutions on aryl carbamates and pyrazol-4-yl methylcarbamates displayed increased toxicity to insecticide-resistant *An. gambiae* and were highly selective for AgAChE compared to hAChE [48]. In addition, PyrimidineTrione Furan-substituted (PTF) compounds have been observed to preferentially bind mutated G119S AChE [223]. Knutsson et al. [224] designed, synthesized and evaluated the biological activity of phenoxyacetamide-based inhibitors of AgAChE and observed that these inhibitors were highly selective for AgAChE compared to hAChE. Also, these inhibitors were effective towards AgAChE with G119S mutation. These studies are pointers to the possibility of chemically modifying current insecticides and developing more species-specific insecticides.

### Combining two or more insecticidal agents in a single product

The concept of having a cocktail of inhibitors in a single insecticide product may slow down the development of insecticidal resistance and be beneficial in killing resistant mosquitoes. An example of this was described in a study that mixed organophosphate and pyrethroid insecticides to obtain a combination that was effective in killing mosquitoes with resistant alleles [225]. Moreover, a new ITN, Interceptor^®^ G2 having a mixture of chlorfenapyr and alphacypermethrin was tested and compared with Interceptor^®^ having only alphacypermethrin and a chlorfenapyr-only net against pyrethroid-resistant *An. gambiae* in experimental field huts [226]. While alphacypermethrin is a pyrethroid insecticide exerting its actions by modulating sodium channels, chlorfenapyr is a pyrrole insecticide that uncouples oxidative phosphorylation thereby preventing ATP synthesis [227]. Camara et al. [226] noted that Interceptor^®^ G2 whether unwashed or washed 20 times significantly killed the mosquitoes by 87% and 82%, respectively, compared to Interceptor^®^ washed or unwashed which resulted in only 10% mortality, while the use of nets treated with chlorfenapyr-only resulted in 92% mortality. Also, Interceptor^®^ G2 unwashed or washed 20 times and chlorfenapyr-only nets greatly inhibited blood-feeding by 42%, 34% and 54%, respectively, unlike Interceptor^®^ which had no significant effect on blood-feeding compared to untreated nets [226]. Additionally, Interceptor^®^ G2 met World Health Organization Pesticide Evaluation Scheme (WHOPES) criteria for further testing and evaluation in phase III study. The mortality rates reported in their study corroborates a previous study, which compared the effect of nets treated with a mixture of chlorfenapyr and alphacypermethrin to those treated with chlorfenapyr only and alphacypermethrin only on mosquito survival [228].

In the study of N’Guessan et al. [228], nets treated with insecticide mixture significantly killed mosquitoes compared to alphacypermethrin-only treated nets (77 vs 30%) and they did not differ significantly from nets treated with chlorfenapyr only (69%). Meanwhile, nets with insecticide mixture induced a higher blood-feeding inhibition on mosquitoes compared to nets treated with alphacypermethrin only (35–51 vs 22%), while no blood-feeding inhibition was evident for nets treated with chlorfenapyr [228]. While both studies affirmed that chlorfenapyr was important for killing pyrethroid-resistant mosquitoes, they did not agree on the contributions of alphacypermethrin and chlorfenapyr to blood-feeding inhibition. The effect of alphacypermethrin on blood-feeding inhibition was suggested by a different study in which ITNs with alphacypermethrin only (MiraNet and MagNet) greatly inhibited blood-feeding compared to untreated nets, despite that it had only limited mortality effect compared to untreated nets [229]. The observed differences in blood-feeding inhibition with alphacypermethrin-only nets in different studies might be due to differences in pyrethroid resistance intensity in the various study areas (Table 2). Despite the differences in blood-feeding inhibition, alphacypermethrin still offers personal protection against mosquito bite. This suggests that alphacypermethrin continues to provide some level of protection even in areas with high pyrethroid resistance intensity. Similar to the study of Camara et al. [226], some other studies suggested that chlorfenapyr also inhibits blood-feeding in mosquitoes. For example, N’Guessan et al. [230] showed that Interceptor^®^ G2, Interceptor^®^ and chlorfenapyr inhibited blood-feeding by 60%, 43% and 57%, respectively. Similarly, in a study on *An. arabiensis*, a blood-feeding inhibition of 76%, 52% and 72% was observed for nets treated with the mixture, alphacypermethrin only and chlorfenapyr only, respectively [231]. Both studies provided evidence that alphacypermethrin and chlorfenapyr, each inhibit the blood-feeding of mosquitoes. Interceptor^®^ G2 may be a replacement for currently available ITNs, thus contributing to the reduction of malaria transmission, considering the increased mortality with their use, and their wash durability (washing Interceptor® G2 only reduced killing efficacy by 2–6%) (Table 2). Although most studies show that mortality rates of mosquitoes did not differ significantly between nets treated with alphacypermethrin and chlorfenapyr mixture and those treated with chlorfenapyr only, combining the insecticides might enhance blood-feeding inhibition and personal protection against bites. Thus, these studies suggested that combining insecticides in a single product could be effective in killing insecticide resistant mosquitoes and reducing their blood-feeding propensity. Further combination of this kind can effectively kill insecticide-resistant mosquitoes and slow down the emergence of insecticide resistance.

**Table 2:** Efficacy of alphacypermethrin and chlorfenapyr mixture in insecticide treated nets

Reference			Camara et al. [226]	Bayili et al. [269]	NʼGuessan et al. [230]	NʼGuessan et al. [228]		Oxborough et al. [231]
Mosquito strain & (location)			*An. gambiae* (*s.s*.) (Côte d’Ivoire)	*An. gambiae* (*s.l*.) (Burkina Faso)	*An. gambiae* (*s.l*.) (Benin)	*An. gambiae* (*s.l*.) (Benin)		*An. arabiensis* (Tanzania)
Pyrethroid resistance intensity (folds)			450.2 (for Alpha). Over 1700 (for deltamethrin)	Over 1000	207 (for Alpha)	207		
Insecticide	Alpha (mg/m^2^)		200 on Interceptor®	200 on Interceptor®	200 on Interceptor®	25		25
	CFP (mg/m^2^)		200	200	200	200		100
	Alpha + CFP (mg/m^2^)		100 + 200 on Interceptor® G2	100 + 200 on Interceptor® G2	100 + 200 on Interceptor® G2	25 + 100	25 + 200	25 + 100
Mortality at 72 h^#^ (%)	Alpha	Unwashed	10^a^	17	20	30		50^f^
		Washed 20 times	11^a^	10	13	nd		nd
	CFP		92^b^	86^d^	76	69^e^		48^f^
	Alpha + CFP	Unwashed	87^b,c^	78^d^	71	75^e^	77^e^	58^f^
		Washed 20 times	82^c^	76^d^	65	nd	nd	nd
Blood-feeding inhibition^##^ (%)	Alpha	Unwashed	ns	26^a,b,c^	57^d^	22^f^		52^g^
		Washed 20 times	ns	15^a^	47^e^	nd		nd
	CFP		54	21^a,c^	43^e^	ns		72^g^
	Alpha + CFP	Unwashed	43	42^b^	60^d^	51	35^f^	76^g^
		Washed 20 times	34	32^b,c^	50^d,e^	nd	nd	nd
Personal protection^###^ (%)	Alpha	Unwashed	57^a,c^	24^d^	62.5^e^	39		nd
		Washed 20 times	47^c^	14^d^	22^f^	nd		nd
	CFP		76^b^	22^d^	36.7^g^	23		nd
	Alpha + CFP	Unwashed	71^a,b^	44^d^	59.2^e^	62	58	nd
		Washed 20 times	60^a^	34^d^	34.4^f,g^	nd	nd	nd

*Abbreviations*: Alpha, alphacypermethrin; CFP, chlorfenapyr; ns, not significant, nd: not determined in the study

For each row (^**#**^, ^**##**^ and ^**###**^), where provided, numbers in the same column (from the same study) sharing a letter superscript do not differ significantly (*P* > 0.05). For resistance status of *An. arabiensis* in Tanzania, percentage mortality of 58 and 76 were observed for lambda cyhalothrin and permethrin (both pyrethroids)

### Combining insecticide with synergists

Another option is the combination of current insecticides with inhibitors to their known detoxifying enzymes. Since oxidative defense greatly impacts insecticide resistance and inhibition of some enzymes involved in oxidative defense increases the sensitivity of the mosquito to insecticide, they can be exploited for development of novel insecticides [232]. Potent insecticide molecules can be combined with inhibitors of detoxification enzymes such as CYP450, GSTs to greatly reduce insecticide resistance. These inhibitor molecules are referred to as synergists. Synergists are chemicals that inhibit metabolic enzymes involved in insecticide detoxification, thereupon allowing the insecticide more time to work, e.g. piperonyl butoxide (PBO) [23]. A practical example of the effect of combining insecticides with synergists, is the increased pyrethroid-susceptibility that was observed when pyrethroids were combined with PBO [23]. In a study by Ketoh et al. [233], higher mortality rates and reduced blood-feeding were observed in mosquitoes that were exposed to pyrethroid-treated nets with PBO compared to those exposed to pyrethroid only treated nets. PBO inhibits cytochrome P450 enzymes, which are key players in insecticide resistance, it also increases cuticular penetration of insecticides [233]. Different pyrethroids have been combined with PBO in ITNs and have been tested for their efficacies in diverse studies. Examples include PermaNet 3.0 (deltamethrin + PBO) [234–236], Olyset® Plus (Permethrin + PBO) [237, 238]. In all these studies, these nets with synergists had higher mortality rates on mosquitoes compared to exposure to their respective insecticide only treated nets, PermaNet 2.0 (deltamethrin only) and Olyset (permethrin only) (Table 3).

**Table 3:** Efficacy of pyrethoids and synergist mixture in insecticide treated nets

Reference	Mosquito strain & (location)	Pyrethroid resistance intensity	Insecticide treated net (ITNs)		Mortality at 72 h^#^ (%)					Blood-feeding inhibition^#^ (%)		Personal protection^###^ (%)	
			Pyrethroid	Pyrethroid + PBO	Untreated nets	Pyrethroid		Pyrethroid + PBO		Pyrethroid	Pyrethroid + PBO	Pyrethroid	Pyrethroid + PBO
						Unwashed	Washed 20 times	Unwashed	Washed 20 times				
Menze et al. [238]	*An. funestus* (Mibellon, Cameroon)	Mortality rates of around 48.88 ± 5.76% for permethrin and around 38.34 ± 5.79% for deltamethrin [270]	Olyset	Olyset Plus	5.4^a^	9.7^a^	nd	25.1	nd	62.37^b^	55.63^b^	83.01^d^	77.35^d^
			PermaNet 2.0	PermaNet 3.0		12.2	nd	30.1	nd	51.36	61.52	70.44^c^	84.90^c^
Oumbouke et al. [239]	*An. gambiae* (*s.s*.) (Côte d’Ivoire)	Mortality rates of 68% for alpha-cypermethrin	MAGNet^®^	VEERALIN®	2.3	29	17.3	51	38.2	35.5	62.7	69	87.1
Toe et al. [271]	*Anopheles gambiae* (*s.l.*) (Burkina Faso)	Mortality rates of < 14% for permethrin and < 33% for deltamethrin	PermaNet 2.0	PermaNet 3.0	9.5	25.9	nd	46.1	nd	nd	nd	nd	nd
			Olyset	Olyset Plus		21.8	nd	36.9	nd	nd	nd	nd	nd
Pennetier et al. [237]	*An. gambiae* (*s.l*.) (Malanville, Benin)		Olyset	Olyset Plus	0	42^a^	36^a^	81^b^	67^b^	82	83	nd	nd
Corbel et al. [235]	*An. gambiae* (*s.s*.) (Vallée du Kou, Burkina Faso)	23% mortality to deltamethrin L1014F kdr mutation (> 80%)	PermaNet 2.0	PermaNet 3.0	4.9	44.4^a^	30.2	78.2	49.3^a^	53.4	72.6	83	86
	(Malanville, Benin)	85% mortality to deltamethrin. 16% L1014F kdr mutation Metabolic resistance (oxidase)			4.2	88.8	70.7^a^	96.7	70.0^a^	90.1	98.7	91.6	99.1
	(Pitoa, Cameroon)	70% mortality to deltamethrin. < 5% L1014F kdr mutation. Metabolic resistance (oxidase + esterase)			12.9	82.9^a^	56.5	93.8	77.9^a^	70.8	46.1	92.3	80.4

*Abbreviations:* nd, not determined in the study; PBO, piperonyl butoxide. For each column (^**#**^, ^**##**^, and ^**###**^), where provided, numbers in the same row (from the same study) sharing a letter superscript do not differ significantly (*P* > 0.05). The insecticide contained in each ITN are as follows: Olyset contains Permethrin; Olyset Plus contains Permethrin + PBO; PermaNet 2.0 contains Deltamethrin; PermaNet 3.0 contains Deltamethrin + PBO; MAGNet^®^ contains Alpha-cypermethrin; VEERALIN^®^ contains Alpha-cypermethrin + PBO

Recently, Oumbouke et al. [239] reported that the use of VEERALIN^®^ nets, an alphacypermethrin PBO synergist net, resulted in a higher mortality rate of mosquitoes (51 vs 29%) and a greater inhibition on blood-feeding (62.6 vs 35.4%) compared to MagNet, an alphacypermethrin-only net. The study suggests that PBO does not only reduce resistance to pyrethroid but, also, potentiates the blood-feeding inhibitory effect of pyrethroids. Meanwhile, loss of efficacy to pyrethroid-based ITNs including Olyset® Plus (low mortality rates of mosquitoes) in *An. funestus* has been reported [240]. These low mortality rates might be due to pyrethroid resistance intensity in study areas as observed in a different study by Corbel et al. [235]. In their study, the study area with the highest pyrethroid resistance intensity had the lowest mortality rate from use of PermaNet 3.0. While this low mortality rate from pyrethroid and synergist-based nets is alarming, these nets still offer a high level of personal protection from mosquito bite (Table 3). Therefore, combining pyrethroid + PBO with an insecticide having a different mode of action for use in ITNs may be advantageous. For example, combining chlorfenapyr, alphacypermethrin and PBO in a single insecticide product may provide a greater advantage by killing pyrethroid-resistant mosquitoes and inhibiting blood-feeding propensity at a higher level. Furthermore, the loss of efficacy to pyrethroid-based ITNs may be due to other resistant mechanisms not addressed by PBO (a cytochrome P450 inhibitor) such as metabolic resistance due to GSTs. Menze et al. [238] observed that *An. funestus* resistant to Olyset or Olyset® Plus due to L119F-GSTe2 mutation had a greater blood-feeding rate compared to mosquitoes with L119 susceptible allele. This mutation was also associated with increased exophily. Consequently, increased blood-feeding rates may greatly enhance the chances of malaria transmission and increased exophily would make the mosquitoes avoid insecticides. Also, their study reported the inability of the synergist, PBO, to prevent metabolic resistance due to GSTs. As a result, incorporating GSTs inhibitors such as diethyl maleate into insecticidal products can reduce insecticide resistance and malaria transmission [238]. Therefore, combining chlorfenapyr, alphacypermethrin (or other pyrethroids), PBO and diethyl maleate (or any other potent GST inhibitor) in a single product may provide an insecticide with better efficacy.

The maintenance of redox homeostasis in *Anopheles* impacts its innate immune response to *Plasmodium*, its survival or longevity as well as its detoxification capacity and consequently its susceptibility to insecticides [145, 165, 232]. NADPH-dependent reducing capacity is a key contributor to the maintenance of redox homeostasis; hence, NADPH concentration is increased during ROS generation. Manipulation of NADPH pools was suggested to affect fecundity and insecticide detoxification capacity of *An. gambiae* [145]. Combining potent insecticide molecules with modulators that can diminish NADPH pools during insecticide application would result in more potent insecticides as insecticide resistance will be greatly reduced.

### Combining insecticides with transmission-blocking agents

In a recent study by Paton et al. [241], *An. gambiae* mosquitoes were exposed to atovaquone (ATQ, an antimalarial which targets the cytochrome b of *Plasmodium*) and other cytochrome b inhibitors such as acequinocyl (ACE) and hydramethylnon (HYD). Exposure to ATQ, HYD and ACE reduced oocyst prevalence by 100%, 63.9% and 64.3%, respectively, relative to controls. As such, exposure of *An. gambiae* mosquitoes to these compounds before *P. falciparum* infection greatly aborted parasite development upon infection. Their study suggests that these cytochrome b inhibitors are suitable agents for transmission-blocking strategies in mosquitoes, as a result preventing malaria transmission. Therefore, combining insecticidal inhibitors of *Anopheles* metabolic proteins with inhibitors of *Plasmodium* metabolic protein (having anti-plasmodial activity) in ITNs or insecticidal spray can serve as a suitable vector control strategy. This combination will prevent transmission by two strategies, either causing mortality of mosquitoes or blocking parasite transmission by clearing the parasite from the midgut of mosquito.

*Anopheles* metabolic proteins can serve as candidates for transmission-blocking vaccines (TBV). For example, amino-peptidase N1 protein (APN) from mosquito midgut was observed to be an antigen that could be targeted by antibodies to prevent *Plasmodium* parasite development. Hence, it has been proposed as a leading TBV candidate [242]. Exposure of mosquitoes to anopheline APN (AnAPN1) monoclonal antibodies efficiently blocked parasite transmission in a dose dependent manner [243].

In addition, the concept of smart sprays has been previously described as chemicals that disrupt interactions that support parasite development, or those that enhance interactions that antagonize parasite development [244]. Therefore, combining insecticides with inhibitors of other metabolic proteins involved in parasite development such as aquaporin 3, trehalose transporter, catalase, KMO, 3HKT, etc., could yield vector control strategies in which resistant mosquitoes that escape insecticides would be unable to transmit malaria due to the action of TBAs. However, inhibition of these proteins must be highly specific for target species.

### Combining insecticides with sterilants

Mitchell & Catteruccia [245] proposed the combination of insecticides with sterilants. With this combination, resistant mosquitoes that escape insecticides would have no progeny due to the sterilant and would not be able to pass their resistance to their progenitors. For instance, combining insecticides with zinc protoporphyrin (ZnPP) and tin protoporphyrin (SnPP), that are inhibitors of heme oxygenase in a single product may offer an advantage of reduced egg-laying in mosquitoes that escape the killing effect of the insecticides. Nevertheless, the safety of these molecules to non-target species must be duly considered. More species-specific heme oxygenase inhibitors may be developed by taking advantage of possible unique features that may be present in anopheline heme oxygenase and absent in non-target species. These inhibitors may serve as sterilants and be used in combination with insecticides.

**Fig. 5:**
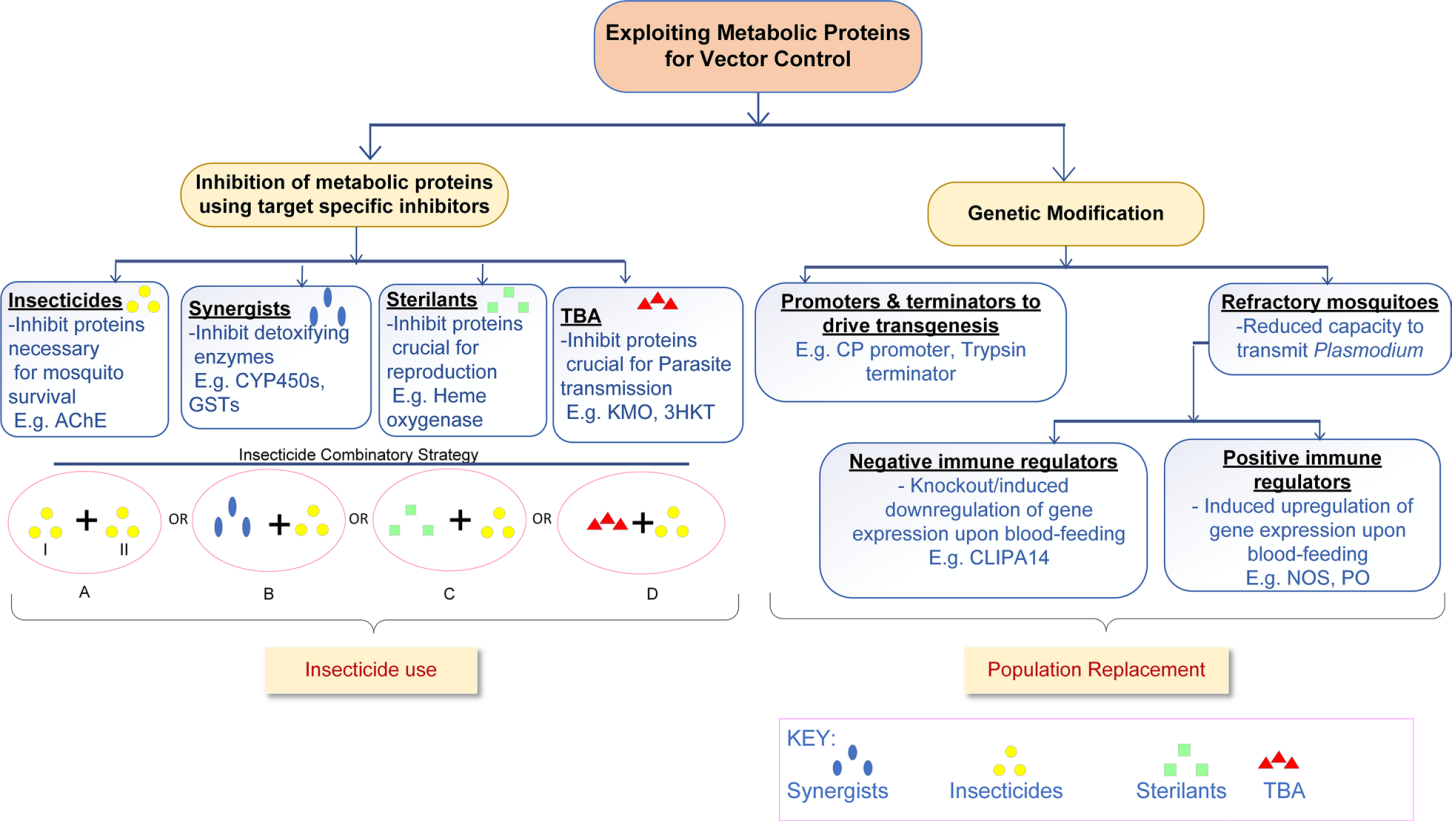
Ways of manipulating metabolic proteins of *Anopheles* for vector control strategies. *Abbreviations*: AChE, acetylcholinesterase; CYP 450, cytochrome P450; GST, glutathione S-transferases; 3HKT, 3-hydroxykynurenine transaminase; KMO, kynurenine 3-monooxygenase; NOS, nitric oxide synthase; PO, phenoloxidase; CP, carboxypeptidase

Whatever method is applied in the development of novel insecticides, the safety to non-target species especially humans must be duly considered. Figure 5 shows a schematic representation, summarizing the various ways metabolic proteins of *Anopheles* could be manipulated for vector control strategies. In addition, the role of some *Anopheles* metabolic proteins in malaria transmission and prevention, as well as the possible intervention strategies that can be achieved by targeting these proteins is shown in Table 4.

**Table 4:** Possible *Anopheles*’ metabolic proteins for vector control strategies based on their role in malaria transmission

Role in malaria transmission	Metabolic protein	Possible intervention strategy
Destruction of *Plasmodium* ookinetes	Chymotrypsin^a^ [136]	Genetic modification: development of refractory mosquitoes with enhanced expression of proteins post blood-feeding. Prevention of peritrophic membrane development
	Trypsin^a^ [135, 136]	
Enhances immune response to *Plasmodium* parasite	Phenylalanine-4-hydroxylase^a^ (PAH) [196]	Genetic modification: development of refractory mosquitoes with increased expression of proteins post blood-feeding
	Nitric oxide synthase^b^ (NOS) [170, 172]	
Enhances *Plasmodium* parasite development or negative regulator of immune response	Carboxypeptidase^a^ [143]	Transmission-blocking agents: inhibition of proteins provides transmission-blocking strategies. Carboxypeptidase can be already targeted using antibodies [143]
	Kynurenine 3-monooxygenase^a^ [179]	
	3-hydroxykynurenine transaminase^a^ [189]	
	Ornithine decarboxylase^a^ [200]	
	Aquaporin 3 [178]	
	Trehalose transporter^c^ [177]	
	Catalase^b^ [158]	
	Vitellogenin^d^ [5]	
	Lipophorin^d^ [5]	
	Apolipophorin^d^ [181, 182]	
Fecundity	Phenylalanine-4-hydroxylase (PAH)^a^ [196]	Sterilants: inhibition may offer sterilizing strategies
	Ornithine decarboxylase^a^ [197]	
	Heme oxygenase [6]	
	Vitellogenin^d^ [5]	
	Catalase^b^ [204]	
Energy production during flight	Trehalase^c^ [156]	Flight inhibitors: inhibition may provide flight inhibition strategies
	Pyrroline-5-carboxylate reductase^a^ [144]	
	Proline oxidase^a^ [144]	
Insecticide resistance	Cytochrome P450 monooxygenases^e^ [238]	Synergists: inhibitors may reverse insecticide resistance
	Glutathione S-transferases^e^ [238]	
Survival or development of mosquitoes	Aquaporin 3 [178]	Insecticides: inhibitors may act as insecticides
	Catalase^b^ [159]	
	3-hydroxykynurenine transaminase^a^ [189]	
	Carbonic anhydrase [52]	
	Arylalkylamine N-acetyltransferases [51]	
	Chorion peroxidase [207]	
	V-ATPases [218, 219]	
	Phosphofructokinase^c^ [50]	

^a^Protein involved in protein/amino acid metabolism

^b^Protein involved in metabolism of reactive oxygen species

^c^Protein involved in carbohydrate metabolism

^d^Protein involved in lipid metabolism

^e^Protein involved in xenobiotic metabolism

## Vector control strategies: genetic modification of metabolism for population replacement or suppression

Population replacement involves substituting *Plasmodium-*susceptible mosquitoes in the wild with laboratory-generated species that are refractory to the parasite, hence incapable of transmitting malaria [246]. This is hinged on genetic modification of the innate immune response of *Anopheles* for enhanced clearance of the parasite. Metabolic proteins that positively regulate the immune response such as NOS, CLIPA8, PO can be genetically modified to increase expression upon blood-feeding. This will ultimately enhance parasite clearance, which will consequently reduce transmission. A study comparing NOS levels in vector and non-vector *An. culicifacies* (i.e. those capable of transmitting disease and those that cannot, respectively) established that elevated midgut levels of inducible NOS upon ingestion of a *Plasmodium* infected blood meal results in effective parasite clearance in non-vector species compared to vector species [247]. Also, inhibition of NOS activity in non-vector species resulted in increased oocyte levels in the mosquito. This study suggests that genetic modification to enhance inducible NOS expression upon blood-feeding can aid parasite clearance and reduce malaria transmission.

Apart from increasing expression of metabolic proteins that positively regulate the immune response, the promoters of some metabolic proteins are significantly activated in specific tissues during a blood meal, e.g. the activation of the carboxypeptidase promoter in the midgut [248] or vitellogenin in the fat tissues [249]. They were induced pbf while apyrase was constitutively expressed in the saliva [250]. These promoters, together with a trypsin terminator, have been used in different studies to drive expression of transgenes [249, 251]. Thus, these promoters and terminator may be used to drive tissue-specific expression of transgenes in *Anopheles* for enhanced immune response and parasite clearance, thereby reducing malaria transmission.

Population suppression involves reducing mosquito population, thereby making them unavailable for malaria transmission. This involves employing genetic techniques to generate a sterile mosquito population. Many metabolic proteins such as heme oxidase or catalase are essential for both fecundity and oviposition. However, these proteins are also essential for the survival and development of mosquitoes. Thus, knockout of these metabolic proteins may induce sterility but will attract a fitness cost making such genetic manipulations unsustainable. As a result, for most population suppression studies, male mosquitoes are made sterile and consequently cannot fertilize the female mosquitoes [252]. To the best of our knowledge, no metabolic protein has been genetically manipulated for the generation of sterile mosquitoes.

Whatever genetic modification is being carried out, fitness cost to the mosquito must be duly considered as the genetically modified mosquitoes must be able to out-compete the wild type.

## Conclusions

*Anopheles* metabolic proteins immensely contribute to the survival of the mosquito and development of *Plasmodium* in the mosquito, and consequently, to malaria transmission. They can be manipulated for vector control strategies. Specific and selective inhibitors can be developed for potential insecticide targets by taking advantage of unique features in targets, thus preventing toxicity to non-target species. Inhibitors discovered to have high insecticidal activity could be used in combinations to slow down the development of resistance to these compounds. Also, these insecticides can be used in combination with synergists, sterilants or TBAs. Likewise, other metabolic proteins that are involved in immune response can be manipulated to produce genetically modified mosquitoes, which are refractory to *Plasmodium*, thereby replacing the susceptible population of mosquitoes. However, the modified species should be able to out-compete the wild-type in nature. Besides this, the safety of genetically-modified strains to the ecosystem must be duly considered. With all these issues considered and put in the right perspective, metabolic proteins of *Anopheles* provide a repertoire for various interventions that would go a long way in curbing malaria transmission.

## Supplementary information

**Additional file 1: Figure S1.** Alignment of the amino acid sequences of AChE from 13 animal species: *Drosophila melanogaster* (DROME), *Tetronarce californica* (TETCF), *Mus musculus* (MOUSE), *Homo sapiens* (HUMAN), *Bos taurus* (BOVIN), *Rattus norvegicus* (RAT), *Caenorhabditis elegans* (CAEEL), *Anopheles stephensi* (ANOST), *An. gambiae* (ANOGA), *Culex pipiens* (CULPI), *An. sinensis* (ANOSI) and *Aedes aegypti* (AEDAE). The positions of the conserved unpaired cysteine and catalytic serine are indicated by a black arrow. The catalytic serine residue is conserved in all the animals. The unpaired cysteine residue is conserved in disease vectors (4–7). This residue is substituted by a leucine residue in *An. stephensi* and *Drosophila* AChE (1–2), phenylalanine residues in mammals, fish and bird AChE (8–13), and a glycine residue in nematode AChE (3). * indicates positions that have single and conserved amino acid residues; : indicates conservation between amino acid residues of strongly similar properties; . indicates conservation between amino acid residues of weakly similar properties.

## Data Availability

Not applicable.

## Abbreviations

ITNs: insecticide-treated nets
IRS: indoor residual spraying
WHO: World Health Organization
PAH: phenylalanine-4-hydroxylase
AChE: acetylcholinesterase
hAChE: human acetylcholinesterase
AgAChE: *An. gambiae* acetylcholinesterase
CYP 450: cytochrome P450
CEs: carboxylesterases
AQP3: aquaporin 3
GSTs: glutathione S-transferases
HPX2: heme peroxidase 2
NOX5: NADPH oxidase 5
NOS: nitric oxide synthase
PO: phenoloxidase
TreT1: trehalose transporter
KMO: kynurenine 3-monooxygenase
PFK: phosphofructokinase
ABC: ATP‐binding cassette
GWAS: genome-wide association studies
pbf: post-blood-feeding
CP: carboxypeptidase
INS: insecticide
SYN: synergist
STER: sterilant
TBA: transmission-blocking agent
GM: genetically modified
H_2_O_2_: hydrogen peroxide
NO: nitric oxide
XA: xanthurenic acid
CAs: carbonic anhydrase
