# Supplementary figures and images for: Anopheles metabolic proteins in malaria transmission, prevention and control: a review
Source: Parasit Vectors. 2020 Sep 10;13:465. doi: 10.1186/s13071-020-04342-5 (PMC7488410; doi:10.1186/s13071-020-04342-5)

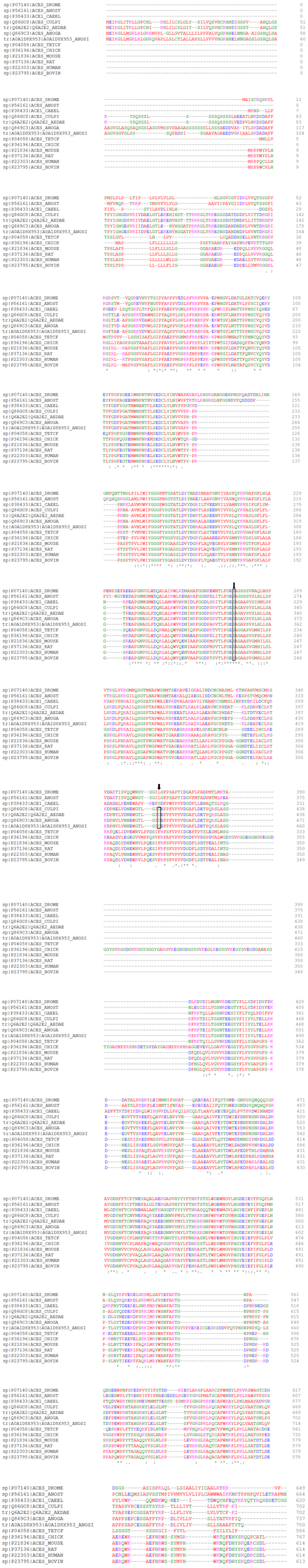

Supplement: Supplementary file 1 — Additional file 1: Figure S1. Alignment of the amino acid sequences of AChE from 13 animal species: Drosophila melanogaster (DROME), Tetronarce californica (TETCF), Mus musculus (MOUSE), Homo sapiens (HUMAN), Bos taurus (BOVIN), Rattus norvegicus (RAT), Caenorhabditis elegans (CAEEL), Anopheles stephensi (ANOST), An. gambiae (ANOGA), Culex pipiens (CULPI), An. sinensis (ANOSI) and Aedes aegypti (AEDAE). The positions of the conserved unpaired cysteine and catalytic serine are indicated by a black arrow. The catalytic serine residue is conserved in all the animals. The unpaired cysteine residue is conserved in disease vectors (4–7). This residue is substituted by a leucine residue in An. stephensi and Drosophila AChE (1–2), phenylalanine residues in mammals, fish and bird AChE (8–13), and a glycine residue in nematode AChE (3). * indicates positions that have single and conserved amino acid residues; : indicates conservation between amino acid residues of strongly similar properties; . indicates conservation between amino acid residues of weakly similar properties. [file 13071_2020_4342_MOESM1_ESM.tif]
